# Ykt6 functionally overlaps with vacuolar and exocytic R-SNAREs in the yeast *Saccharomyces cerevisiae*

**DOI:** 10.1016/j.jbc.2024.107274

**Published:** 2024-04-06

**Authors:** Hayate Watanabe, Shingo Urano, Nozomi Kikuchi, Yurika Kubo, Ayumi Kikuchi, Katsuya Gomi, Takahiro Shintani

**Affiliations:** Department of Agricultural Chemistry, Graduate School of Agricultural Science, Tohoku University, Sendai, Japan

**Keywords:** SNARE proteins, membrane trafficking, membrane fusion, autophagy, *Saccharomyces cerevisiae*, vacuole, plasma membrane

## Abstract

The soluble *N*-ethylmaleimide-sensitive factor attachment protein receptor (SNARE) complex forms a 4-helix coiled-coil bundle consisting of 16 layers of interacting side chains upon membrane fusion. The central layer (layer 0) is highly conserved and comprises three glutamines (Q) and one arginine (R), and thus SNAREs are classified into Qa-, Qb-, Qc-, and R-SNAREs. Homotypic vacuolar fusion in *Saccharomyces cerevisiae* requires the SNAREs Vam3 (Qa), Vti1 (Qb), Vam7 (Qc), and Nyv1 (R). However, the yeast strain lacking *NYV1* (*nyv1Δ*) shows no vacuole fragmentation, whereas the *vam3Δ* and *vam7Δ* strains display fragmented vacuoles. Here, we provide genetic evidence that the R-SNAREs Ykt6 and Nyv1 are functionally redundant in vacuole homotypic fusion *in vivo* using a newly isolated *ykt6* mutant. We observed the *ykt6-104* mutant showed no defect in vacuole morphology, but the *ykt6-104 nyv1Δ* double mutant had highly fragmented vacuoles. Furthermore, we show the defect in homotypic vacuole fusion caused by the *vam7-Q284R* mutation was compensated by the *nyv1-R192Q* or *ykt6-R165Q* mutations, which maintained the 3Q:1R ratio in the layer 0 of the SNARE complex, indicating that Nyv1 is exchangeable with Ykt6 in the vacuole SNARE complex. Unexpectedly, we found Ykt6 assembled with exocytic Q-SNAREs when the intrinsic exocytic R-SNAREs Snc1 and its paralog Snc2 lose their ability to assemble into the exocytic SNARE complex. These results suggest that Ykt6 may serve as a backup when other R-SNAREs become dysfunctional and that this flexible assembly of SNARE complexes may help cells maintain the robustness of the vesicular transport network.

Vesicular trafficking in the endomembrane system depends on the budding of vesicles from the donor membrane and their fusion with the acceptor membrane. To maintain proper membrane flow in a vesicular transport network, vesicles must be selectively targeted to the acceptor membrane for fusion (1). Various Rab family GTPases and phosphatidylinositol variants play pivotal roles in recruiting effectors (tethering factors) to bridge donor and acceptor membranes. After tethering between the vesicles and the target membrane, different soluble *N*-ethylmaleimide-sensitive factor attachment protein receptor (SNARE) proteins located on the separated membranes assemble into a *trans*-SNARE complex, thereby driving the fusion of lipid bilayers. SNARE proteins are typically anchored to the membrane *via* their C-terminal transmembrane domain and have a heptad repeat of 60 to 70 amino acids in their cytosolic domain, called the SNARE motif, which assembles a 4-helix coiled-coil bundle in the *trans*-SNARE complex. SNAREs are classified by sequence similarity into Q-SNAREs and R-SNAREs, which contain glutamine (Q) and arginine (R) residues, respectively, in the center of the SNARE motif called the “0-layer” (2). Q-SNAREs are further classified as Qa-, Qb-, and Qc-SNAREs based on their structural features (3). The assembly of intrinsic QabcR-SNARE complexes, facilitated by a specific Sec1/Munc18-family protein, serves as the final checkpoint to ensure the fidelity of the vesicular transport network (4). The yeast *Saccharomyces cerevisiae* has 24 SNAREs, each localized to at least one specific organelle and assigned to a specific fusion step(s) through genetic and cell biological analyses (1). *In vitro* reconstitution of QabcR-SNARE complexes and a fusion assay with proteoliposomes endorsed many cognate SNARE pairings occurring *in vivo*. In contrast, the *in vitro* assembly of some SNAREs is often relatively promiscuous, especially endosomal, vacuolar, and plasma membrane SNAREs (5, 6, 7). However, whether this promiscuity is biologically important remains unclear.

The lysosome/vacuole is a membrane-bound lytic compartment in which hydrolytic enzymes encounter macromolecules intended for degradation. In *S*. *cerevisiae*, multiple transport routes converge at the vacuole, such as the Golgi-to-vacuole and endosome-to-vacuole pathways and macroautophagy (hereafter referred to as autophagy). The vacuoles, which are fragmented during cell division or in response to the environmental stimuli such as osmotic shock, also use homotypic fusion to maintain their structure and functions. In these pathways, Ypt7, the Mon1-Ccz1 complex, and homotypic fusion and vacuole protein sorting (HOPS) complex are commonly used as a Rab GTPase, a guanine nucleotide exchange factor for Ypt7, and a tethering factor, respectively, to assemble a *trans*-SNARE complex (8, 9). However, these pathways do not always use the same set of SNAREs. *In vitro* reconstitution experiments have revealed that homotypic vacuolar fusion utilizes Vam3 (Qa), Vti1 (Qb), Vam7 (Qc), and Nyv1 (R) as SNAREs, where Nyv1 is substituted with Ykt6 in autophagosome-vacuole fusion (6, 10, 11, 12). Since all fusion processes involving these SNARE combinations require Ypt7 and HOPS, they may be subclasses of SNARE complexes that operate *via* a common mechanism. Strains with deleted genes involved in homotypic vacuolar fusion, such as *vam3Δ*, *vam7Δ*, *ypy7Δ*, *mon1Δ*, *ccz1Δ*, *vam6Δ* (HOPS), and *vps41Δ* (HOPS), have fragmented vacuoles (13, 14, 15, 16, 17). However, the *nyv1Δ* strain lacks vacuole fragmentation (18, 19). Although *in vitro* fusion experiments have suggested that the loss of Nyv1 function is compensated for by Ykt6 (10, 20), little is known about the functional overlap between Ykt6 and Nyv1 *in vivo*.

Here, we show that Ykt6 and Nyv1 are functionally redundant *in vivo* in vacuole homotypic fusion using newly isolated *ykt6* mutants. Isolated *ykt6* mutants are temperature-sensitive for autophagosome-vacuole fusion but do not show any defects in vacuole morphology. Deleting the *NYV1* gene in these mutants resulted in highly fragmented vacuoles, indicating that *YKT6* and *NYV1* have strong genetic interactions. Complementary 0-layer mutations have confirmed that Nyv1 is exchangeable for Ykt6 in the vacuole SNARE complex to maintain homotypic vacuole fusion. Surprisingly, Ykt6 likely assembles with exocytic Q-SNAREs instead of Snc1 and its paralog Snc2, which are intrinsic exocytic R-SNAREs, when Snc1 and Snc2 lose their ability to assemble the exocytic SNARE complex. Finally, we also discussed the functional overlaps between Ykt6 and other R-SNAREs.

## Results

### Generation of new ykt6 mutant strains with defects in the fusion of autophagosomes and vacuoles

**Figure 1:**
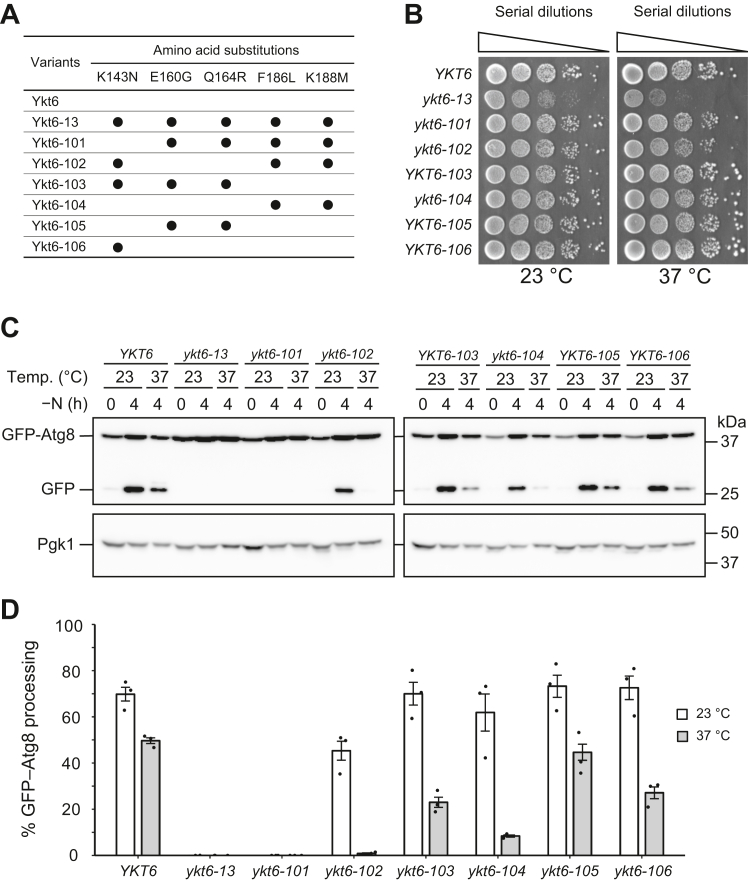
**Analysis of cell growth and autophagic flux in newly generated *ykt6* mutants.***A*, summary of amino acid substitutions in Ykt6 variants. *Black circles* indicate the locations of amino acid substitution in each variant. *B*, the WT (YSU34), *ykt6-13* (YSU37), *ykt6-101* (YTS856), *ykt6-102* (YTS858), *YKT6-103* (YTS860), *ykt6-104* (YTS862), *YKT6-105* (YTS864), and *YKT6-106* (YTS866) strains were grown in YPD medium at 23 °C. Cells equivalent to 10^0^ to 10^−4^*A*_600_ units were spotted on YPD agar medium and incubated at 23 °C and 37 °C for 3 days, respectively. *C*, the WT and a series of *ykt6* variants expressing GFP–Atg8 were grown in SCD−Ura medium to the early log phase at 23 °C and resuspended in SD(−N) medium for further incubation at 23 °C and 37 °C for 4 h. The total cell lysates were subjected to Western blot analysis with anti-GFP and anti-3-phosphoglycerate kinase (Pgk1) antibodies. *D*, the experiments in *C* were repeated three times and the signal intensity was quantified to calculate the rate of GFP–Atg8 cleavage as autophagic flux. *Dots* and *error bars* indicate the actual values and the standard error of the mean, respectively. SCD, synthetic casamino acid dextrose; SD, synthetic dextrose; YPD, yeast extract peptone dextrose.

To analyze the function of Ykt6 in homotypic vacuolar fusion *in vivo*, we focused on the *ykt6-13* mutation, which confers a defect in membrane fusion between transport vesicles and the vacuole in the AP-3 (Golgi-to-vacuole), carboxypeptidase Y (CPY, endosome-to-vacuole), and cytoplasm-to-vacuole targeting pathways (21). Although the *ykt6-13* strain shows temperature-sensitive growth inhibition, all vacuolar transport pathways are inhibited even at permissive temperatures, resulting in a constitutive loss of normal vacuole biogenesis, which hampers the analysis of the functional redundancy between Nyv1 and Ykt6. Recent *in vitro* studies have suggested that Ykt6 is the only R-SNARE required for the fusion between autophagosomes and vacuoles during autophagy (11, 12), prompting us to evaluate the function of Ykt6 derivatives by analyzing the autophagy process. The Ykt6-13 protein has amino acid substitutions at five residues in its SNARE domain. We speculated that these substitutions might confer severe defects in transport pathways other than membrane fusion at the vacuole. Therefore, we systematically restored these mutated residues to their original ones (Fig. 1*A*) and examined their effects on growth and autophagy. To assess cell growth, yeast cells expressing the WT or mutant Ykt6 proteins were subjected to a spot assay on yeast extract peptone dextrose (YPD) plates at 23 °C and 37 °C. As shown in a previous report (21), the *ykt6-13* strain exhibited a severe growth defect at 37 °C. In contrast, the growth of the newly constructed strains was equivalent to that of the *YKT6* strain, although *ykt6-101* and *ykt6-102* showed slightly delayed growth at 37 °C (Fig. 1*B*). Next, we performed a GFP–Atg8 processing assay, which can evaluate autophagic flux by monitoring the release of the GFP moiety from GFP–Atg8 in the vacuole (22, 23). The cells were grown at 23 °C, transferred to a starvation medium for further incubation at 23 °C or 37 °C, and the total cell lysates were subjected to Western blot analysis with anti-GFP antibodies. In WT cells, a free GFP band was observed upon starvation at both temperatures (Fig. 1, *C* and *D*). In contrast, no free GFP band was observed in *ykt6-13* cells under either condition (Fig. 1, *C* and *D*). These results indicate that autophagic flux was constitutively blocked in *ykt6-13* cells. Similarly, no GFP–Atg8 processing was observed in the *ykt6-101* cells at either temperature (Fig. 1, *C* and *D*). Notably, the *ykt6-102* and *ykt6-104* strains were temperature-sensitive for autophagy, with processing confirmed at 23 °C but greatly decreased at 37 °C (Fig. 1, *C* and *D*). In contrast, autophagy was almost normal in the *YKT6-103*, *YKT6-105*, and *YKT6-106* strains (Fig. 1, *C* and *D*).

**Figure 2:**
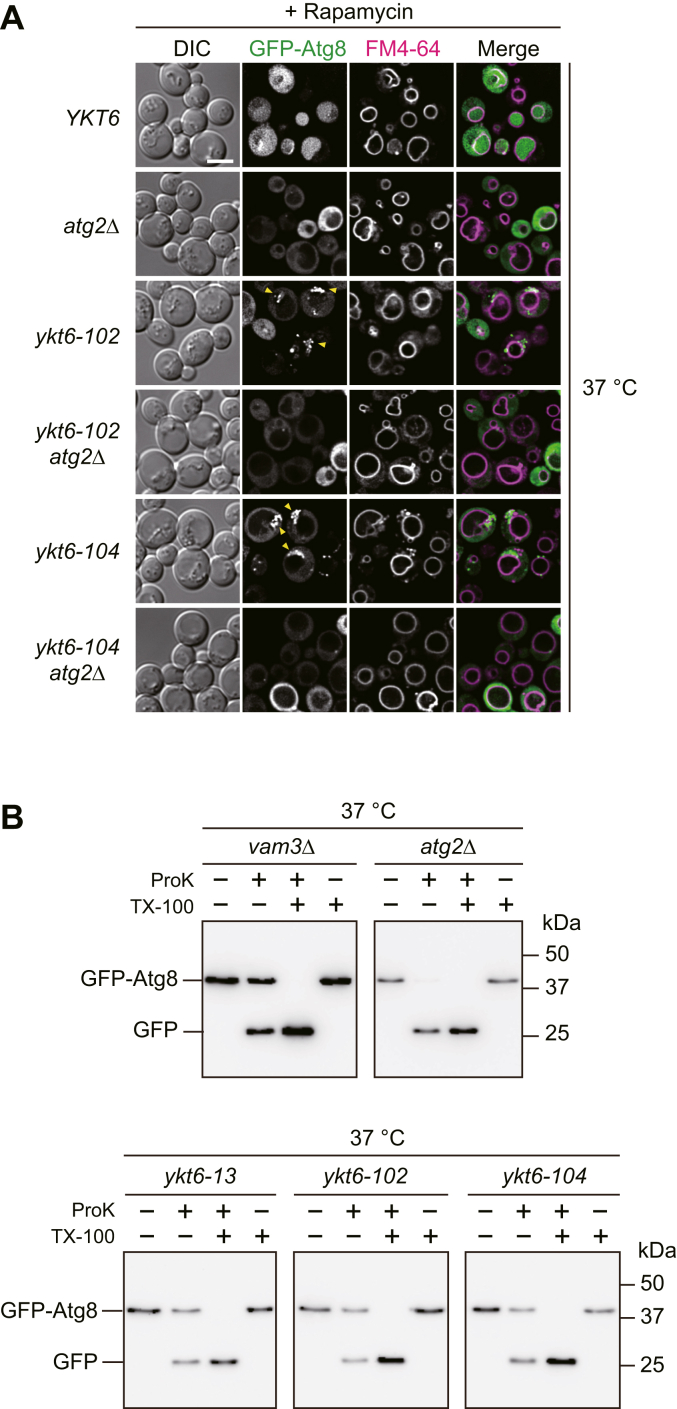
**The fusion of autophagosomes with vacuoles is defective in the *ykt6-102* and *ykt6-104* mutants.***A*, the WT (YSU34), *atg2Δ* (YTS837), *ykt6-102* (YTS858), *ykt6-102 atg2Δ* (YTS854), *ykt6-104* (YTS862), and *ykt6-104 atg2Δ* (YTS843) strains expressing GFP–Atg8 were grown in SCD−Ura medium to the early log phase at 23 °C. The cells were then stained with FM 4-64 for 15 min and resuspended in fresh SCD−Ura medium containing 0.2 μg/ml rapamycin to induce autophagy. The cells were further incubated at 37 °C for 2 h and observed *via* fluorescence microscopy. *Yellow arrowheads* indicate the accumulation of autophagosomes out of the vacuole. The scale bar represents 5 μm. *B*, the GFP-Atg8 protease protection assay. The *vam3Δ*, *atg2Δ* (YTS837), *ykt6-13* (YSU37), *ykt6-102* (YTS858), and *ykt6-104* (YTS862) strains expressing GFP–Atg8 were grown in SCD−Ura medium to the early log phase at 23 °C. Then, the cells were shifted to SD(−N) medium and incubated at 37 °C for 3 h. The membrane fractions were collected by treating with proteinase K (ProK) in the presence or absence of 0.2% Triton X-100 (TX-100) and analyzed *via* Western blotting using anti-GFP antibodies. FM 4-64, N-(3-triethylammoniumpropyl)-4-(6-(4-(diethylamino) phenyl) hexatrienyl) pyridinium dibromide; SCD, synthetic casamino acid dextrose; SD, synthetic dextrose.

Next, we observed the localization of GFP–Atg8 to understand which autophagic process was blocked by the *ykt6-102* and *ykt6-104* mutations. Atg8 is incorporated into autophagosomes and transported to the vacuoles so that GFP–Atg8 accumulates on autophagosomes when their fusion to the vacuole is inhibited (24, 25, 26). WT and mutant cells grown in a nutrient-rich medium at 23 °C were transferred to the same medium containing rapamycin and incubated for another 2 h at 37 °C to induce autophagy. In WT cells, GFP–Atg8 signals were dispersed throughout the vacuole lumen, indicating the induction of autophagy (Fig. 2*A*). In contrast, a cluster of multiple GFP–Atg8 puncta was observed in the cytoplasm in *ykt6-102* and *ykt6-104* cells. When *ATG2*, a gene essential for autophagosome formation, was deleted, the puncta clusters disappeared (Fig. 2*A*). Next, we biochemically examined autophagosome accumulation in *ykt6-13*, *ykt6-102*, and *ykt6-104* cells using a GFP–Atg8 protease protection assay (27). Membrane fractions were collected from the cell extracts of each strain starved at 37 °C and treated with exogenously added protease in the presence or absence of a detergent. Western blot analysis with anti-GFP antibody revealed that a portion of GFP–Atg8 was protected from exogenously added protease without detergent in *ykt6-13*, *ykt6-102*, and *ykt6-104* cells as well as in *vam3Δ* cells, which is known to accumulate autophagosomes, but not in *atg2Δ* cells (Fig. 2*B*). Taken together, these results suggest that the fusion of autophagosomes with vacuoles is inhibited by *ykt6-102* and *ykt6-104* mutations, resulting in the accumulation of autophagosomes in *ykt6-102* and *ykt6-104* cells.

### Characterization of ykt6 mutants for vacuole morphology and vacuolar protein sorting

**Figure 3:**
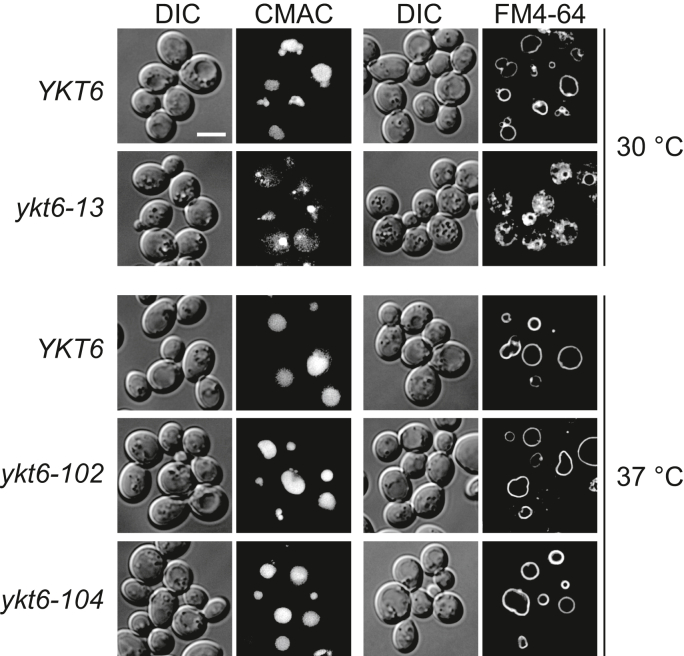
**Vacuolar morphology of the *ykt6* mutant strains.** The WT (YSU34), *ykt6-13* (YSU37), *ykt6-102* (YTS858), and *ykt6-104* (YTS862) strains were grown in SCD medium to the early log phase at 30 °C or 37 °C. The cells were stained with CMAC and observed *via* fluorescence microscopy to observe the vacuole. To stain the vacuolar membrane, the cells were incubated in SCD medium containing FM 4-64 for 15 min at the same temperature, resuspended in fresh SCD medium for further incubation for 90 min at the same temperature, then observed using fluorescence microscopy. The scale bar represents 5 μm. CMAC, 7-amino-4-chloromethylcoumarin; FM 4-64, N-(3-triethylammoniumpropyl)-4-(6-(4-(diethylamino) phenyl) hexatrienyl) pyridinium dibromide; SCD, synthetic casamino acid dextrose.

The *ykt6-102* and *ykt6-104* mutants were then evaluated for vacuole morphology and vacuolar protein sorting. To examine the vacuole morphology of the *ykt6* mutants, WT and *ykt6* mutant cells were grown at 37 °C and labeled with the styryl dye N-(3-triethylammoniumpropyl)-4-(6-(4-(diethylamino) phenyl) hexatrienyl) pyridinium dibromide (FM 4-64) or 7-amino-4-chloromethylcoumarin (CMAC). Fluorescence microscopy revealed that the vacuoles of *ykt6-102* and *ykt6-104* cells appeared normal and indistinguishable from those of the WT strain (Fig. 3). In contrast, *ykt6-13* cells grown at 30 °C had multiple FM 4-64-positive structures with various sizes and a relatively small CMAC-positive round-shaped structure (∼2 μm in diameter). These results suggest that the *ykt6-102* and *ykt6-104* strains have few defects in homotypic vacuole fusion.

**Figure 4:**
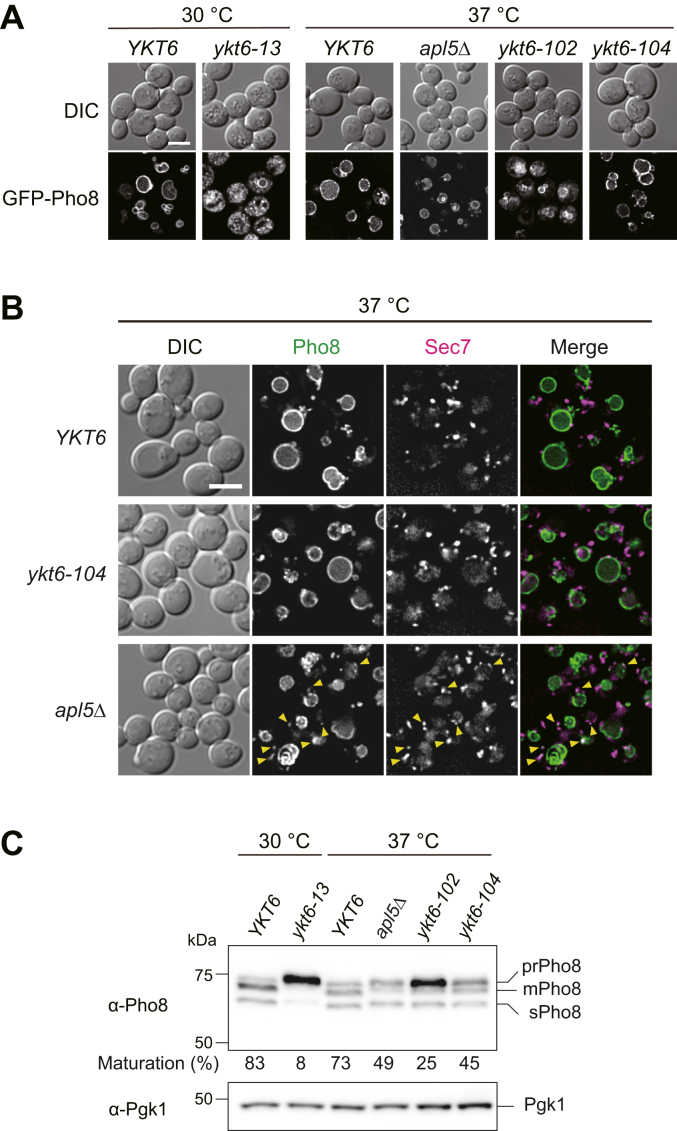
**Characterization of the AP-3 pathway in *ykt6* mutant strains.***A*, the WT (YHW221), *ykt6-13* (YHW233), *apl5Δ* (YHW491), *ykt6-102* (YHW223), and *ykt6-104* (YHW225) strains expressing GFP–Pho8 were grown in SCD−Ura medium to the early log phase at 30 °C or 37 °C and observed *via* fluorescence microscopy. The scale bar represents 5 μm. *B*, the WT (YHW581), *ykt6-104* (YHW582), and *apl5Δ* (YHW585) strains expressing GFP-Pho8 and Sec7-mCherry were grown in SCD−Ura medium to the early log phase at 37 °C and observed *via* fluorescence microscopy. *Yellow arrowheads* indicate colocalization of GFP-Pho8 and Sec7-mCherry. The scale bar represents 5 μm. *C*, the WT (YSU34), *ykt6-13* (YSU37), *apl5Δ*, *ykt6-102* (YTS858), and *ykt6-104* (YTS862) strains were grown in SCD medium to the early log phase at 30 °C or 37 °C, and the cell lysates were subjected to Western blot analysis with anti-Pho8 antibodies. The positions of the precursor form (prPho8; nonvacuole), the mature form (mPho8; vacuole), and the soluble form (sPho8; vacuole) of alkaline phosphatase are shown. The ratio of precursor form to total Pho8 (the precursor + mature form + soluble form) was calculated with the data from three independent experiments, the average of which is shown below the Pho8 blot. SCD, synthetic casamino acid dextrose.

The AP-3 (Golgi-to-vacuole) pathway was evaluated by examining the localization and proteolytic processing of the alkaline phosphatase Pho8. WT and *ykt6* mutant strains expressing GFP-tagged Pho8 were grown at 37 °C (or 30 °C for the *ykt6-13* strain) and observed *via* fluorescence microscopy. GFP–Pho8 was detected at the vacuole membrane in *ykt6-104* cells and in WT cells (Fig. 4*A*). In *ykt6-104* cells, some GFP–Pho8-positive dots were also observed in the cytoplasm, but they did not colocalize with the late Golgi marker Sec7–mCherry (Fig. 4*B*). In contrast, GFP–Pho8 was detected as several tiny dots in the cytoplasm in addition to vacuole membrane localization in *ykt6-102* cells, and vacuole membrane localization was almost absent in *ykt6-13* cells (Fig. 4*A*). Some cargo proteins of the AP-3 pathway including Pho8 reach to the vacuole *via* the CPY pathway when the AP-3 pathway is deficient (28, 29, 30). Therefore, we compared the localization of GFP–Pho8 in *ykt6* mutant strains and *apl5Δ* strain, defective in the AP-3 pathway. When the *apl5Δ* cells were grown at 37 °C, in addition to the vacuolar membrane localization, GFP–Pho8 was observed as several cytoplasmic dots (Fig. 4*A*), some of which colocalized with Sec7–mCherry (Fig. 4*B*). GFP–Pho8 also localized to the intralumenal membranes of the vacuole (Fig. 4, *A* and *B*) in the *apl5Δ* cells as observed in the strain lacking Apl6, another subunit of AP-3 adapter complex (31), although no intralumenal membranes were not observed in the *ykt6-102* and *ykt6-104* strains. These results suggested that the partial defects in the vacuolar transport of Pho8 in *ykt6* mutants may not be due to the blockage of the AP-3 pathway. Consistently, Pho8 processing was slightly affected in *ykt6-104* cells than in WT cells; however, the precursor form accumulated in *ykt6-102* cells, although not as much as in the *ykt6-13* strain (Fig. 4*C*). Taken together, these results suggest that vacuolar transport of Pho8 occurs *via* the AP-3 pathway in the *ykt6-102* and *ykt6-104* strains, albeit with partial defects.

**Figure 5:**
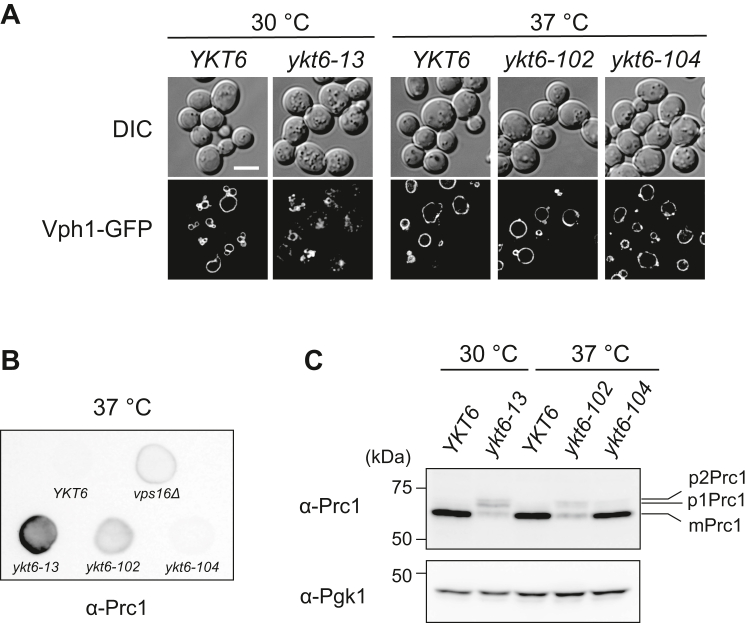
**Characterization of the CPY pathway in *ykt6* mutant strains.***A*, the WT (YHW227), *ykt6-13* (YHW235), *ykt6-102* (YHW229), and *ykt6-104* (YHW231) strains expressing Vph1–GFP were grown to the early log phase at 30 °C or 37 °C and observed using fluorescence microscopy. The scale bar represents 5 μm. *B*, the WT (YSU34), *vps16Δ*, *ykt6-13* (YSU37), *ykt6-102* (YTS858), and *ykt6-104* (YTS862) strains were grown in YPD medium to the early log phase at 23 °C. Then, four *A*_600_ units of cells were spotted onto a nitrocellulose membrane on an SD agar medium containing Ura/Leu/His/Lys and incubated for 20 h at 37 °C. After removing the cells, Prc1 secretion on the nitrocellulose membrane was detected using anti-Prc1 antibodies. *C*, the WT (YSU34), *ykt6-13* (YSU37), *ykt6-102* (YTS858), and *ykt6-104* (YTS862) strains were grown in SCD medium to the early log phase at 30 °C or 37 °C and the cell lysates were subjected to Western blot analysis with anti-Prc1 antibodies. The positions of p1Prc1 (ER), p2Prc1 (Golgi), and mPrc1 (vacuole) are shown. CPY, carboxypeptidase Y; ER, endoplasmic reticulum; SCD, synthetic casamino acid dextrose; SD, synthetic dextrose; YPD, yeast extract peptone dextrose.

CPY, which is encoded by the *PRC1* gene, and the vacuolar ATPase V0 domain are transported from the late-Golgi compartment to the vacuole *via* the endosome (32, 33). Fluorescence microscopy revealed that GFP-tagged Vph1, a subunit of the vacuolar ATPase V0 domain, was normally localized to the vacuolar membrane in *ykt6-102* and *ykt6-104* cells, as in the WT, but was severely affected by *ykt6-13* mutation (Fig. 5*A*). A previous report showed that *ykt6-13* mutation causes Prc1 secretion, indicating a defect in endosomal sorting (21). To clarify whether the *ykt6-102* and *ykt6-104* strains had defects in endosomal sorting, the secretion of Prc1 was examined using colony immunoblotting. As expected, the *ykt6-13* cells missorted Prc1 outside the cells, as well as the strain with a deletion of *VPS16*, the gene encoding a common member of the class C core vacuole/endosome tethering (CORVET), and HOPS complexes essential for membrane docking and fusion at the Golgi-to-endosome and endosome-to-vacuole protein transport stages, respectively (Fig. 5*B*) (34). Interestingly, Prc1 was secreted by the *ykt6-102* strain even though Vph1–GFP was normally localized to the vacuolar membrane (Fig. 5*B*). In contrast, Prc1 secretion was very limited in the *ykt6-104* and WT strains (Fig. 5*B*). Consistently, Western blotting revealed that most Prc1 was detected as a mature form in the *ykt6-104* strain, similar to the WT strain (Fig. 5*C*). Taken together, these results suggest that the *ykt6-104* strain has normal vacuoles and that the *ykt6-102* strain had some defects in vacuolar protein sorting.

### Ykt6 and Nyv1 are functionally exchangeable in homotypic vacuole fusion

**Figure 6:**
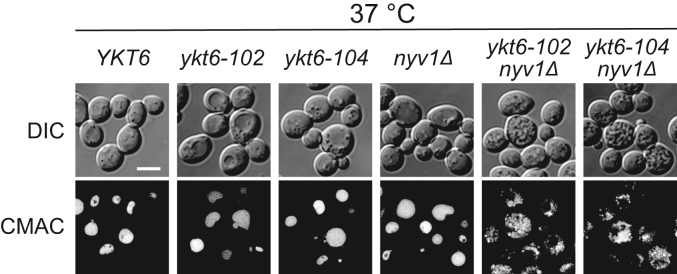
**Vacuolar morphology of the *ykt6 nyv1* double mutants.** The WT (YSU34), *ykt6-102* (YTS858), *ykt6-104* (YTS862), *nyv1Δ* (YTS868), *ykt6-102 nyv1Δ* (YTS872), and *ykt6-104 nyv1Δ* (YTS876) strains were grown in YPD medium to the early log phase at 37 °C. The vacuoles were stained with CMAC and observed using fluorescence microscopy. The scale bar represents 5 μm. CMAC, 7-amino-4-chloromethylcoumarin; YPD, yeast extract peptone dextrose.

Next, we examined the genetic interaction between *YKT6* and *NYV1* using the newly constructed *ykt6* mutants for homotypic vacuole fusion. Accordingly, we deleted the *NYV1* gene in the *ykt6-102* and *ykt6-104* strains and compared their vacuole morphology to the single *nyv1Δ* strain. CMAC staining revealed that the double mutants exhibited multiple dots, representing a typical morphology of the fragmented vacuole as seen in *vam7Δ* cells; *nyv1Δ* cells showed normal vacuoles (Fig. 6). These results suggested that Ykt6 and Nyv1 are functionally redundant during homotypic vacuole fusion.

**Figure 7:**
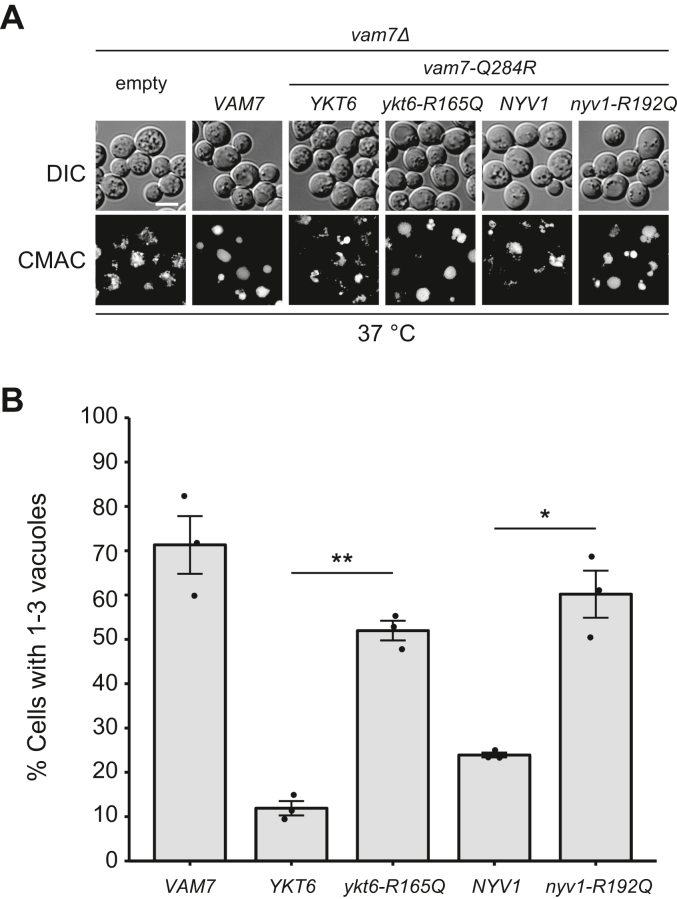
**Vacuolar morphology of the *vam7-Q284R* mutant expressing R-SNARE with a complementary 0-layer mutation.***A*, the *vam7-Q284R* strain expressing either an empty vector, Ykt6, Ykt6-R165Q, Nyv1, or Nyv1-R192Q, was grown in SC−His/Leu medium to the early log phase at 37 °C. The cells were then stained with CMAC and observed using fluorescence microscopy. The scale bar represents 5 μm. *B*, the number of cells with one to three vacuoles was counted in the fluorescence microscopy images observed in the same manner as in *A*. For each strain and each experiment, at least 100 cells containing fluorescent vacuoles were quantified using fluorescence microscopy. *Dots* and *error bars* indicate the actual values and the standard error of the mean for three independent experiments, respectively. Statistical analysis was conducted using Welch's *t* test. ∗*p* < 0.05, ∗∗*p* < 0.01. CMAC, 7-amino-4-chloromethylcoumarin; SNARE, soluble N-ethylmaleimide-sensitive factor attachment protein. SC, synthetic complete.

Next, we examined whether Ykt6 directly interacts with vacuolar SNAREs in homotypic vacuole fusion *in vivo*. We took advantage of complementary SNARE mutations that enabled us to identify possible combinations of SNARE complexes. Upon membrane fusion, SNAREs form 4-helix coiled-coils within their SNARE domains. Although the contact faces in this bundle of SNARE complexes generally comprise nonpolar amino acids, three glutamines (Q) and one arginine (R) localized in the middle of the SNARE domain form the conserved ionic 0-layer. The Q-to-R substitution in the 0-layer of a Q-SNARE, yielding the 2Q:2R complex, results in the loss of SNARE function both *in vivo* and *in vitro*. When the 3Q:1R ratio is restored by an additional R-to-Q substitution in the corresponding R-SNARE, the function of the SNARE complex is recovered in many cases (35, 36, 37, 38, 39). To perform a complementary substitution assay, we constructed a *vam7Δ* strain expressing Vam7-Q284R, whose 0-layer glutamine residue was substituted with arginine. The *vam7-Q284R* strain (*vam7Δ* strain expressing Vam7-Q284R) showed a temperature-sensitive phenotype; it had normal vacuoles at 23 °C but fragmented vacuoles at 37 °C (Fig. S1). Next, we expressed WT and R-to-Q-substituted Ykt6 (Ykt6-R165Q) in the *vam7-Q284R* strain and stained the vacuoles with CMAC. Fluorescence microscopy observations revealed that Ykt6-R165Q recovered vacuole morphology, but the WT Ykt6 did not (Fig. 7), suggesting that Ykt6-R165Q could restore the 3Q:1R ratio in the Vam3-Vti1-Vam7-Ykt6 complex to function as a vacuole SNARE. Similarly, Nyv1-R192Q restored the loss of homotypic vacuole fusion in the *vam7-Q284R* strain (Fig. 7). These results suggested that Ykt6 and Nyv1 act redundantly as vacuole R-SNAREs for homotypic vacuole fusion. Ykt6-R165Q restored autophagic flux in the *vam7-Q284R* strain, whereas Nyv1-R192Q did not (Fig. S2), confirming that Ykt6, but not Nyv1, functions as an R-SNARE for the fusion between autophagosomes and vacuoles.

**Figure 8:**
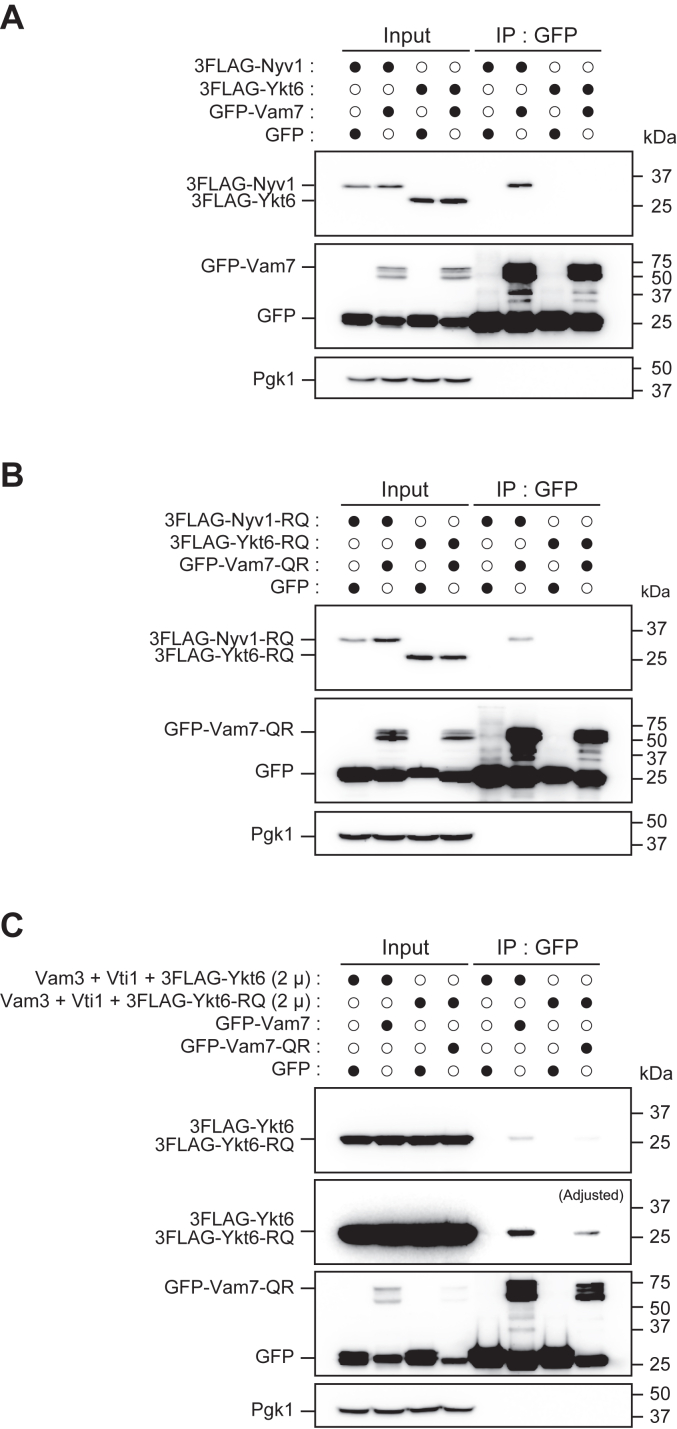
**Analysis of physical interactions of Vam7 with R-SNAREs, Nyv1 and Ykt6, by coimmunoprecipitation experiments.***A*, total lysate from the WT cells (BY4742) expressing either 3FLAG−Nyv1 or 3FLAG−Ykt6 from low-copy plasmids and either expressing GFP or GFP−Vam7 were used for immunoprecipitation with anti-GFP antibody, and the immunoprecipitates were analyzed by Western blot with anti-FLAG, anti-GFP, and anti-Pgk1 antibodies. *Closed circles* indicate plasmids retained in the cells used for analyses. *B*, total lysate from the WT cells expressing either 3FLAG−Nyv1-R192Q or 3FLAG−Ykt6-R165Q from low-copy plasmids and either expressing GFP or GFP−Vam7-Q284R were used for analysis as in *A*. *C*, coimmunoprecipitation experiments were performed as in *A* and *B* using WT cells overexpressing *VAM3*, *VTI1*, and *3FLAG − YKT6* or *3FLAG−ykt6-R165Q* from a single multicopy plasmid (2 μm plasmid). SNARE, soluble N-ethylmaleimide-sensitive factor attachment protein.

To further verify that Ykt6 physically interacts with the vacuolar Q-SNARE, coimmunoprecipitation experiments were performed. The centromeric plasmid (low copy number) containing *3FLAG − NYV1* or *3FLAG − YKT6* was introduced to the WT strain expressing GFP-tagged Vam7, and GFP−Vam7 was immunoprecipitated with anti-GFP antibody. 3FLAG−Nyv1 was noticeably coprecipitated with GFP−Vam7, but 3FLAG−Ykt6 was not (Fig. 8*A*). We also found that GFP−Vam7-Q284R and 3FLAG−Nyv1-R192Q maintained physical interaction (Fig. 8*B*). When SNARE proteins were expressed from the multicopy plasmid encoding *VAM3*, *VTI1*, and *3FLAG-YKT6*, GFP−Vam7 coprecipitated with 3FLAG−Ykt6 (Fig. 8*C*). In this condition, the physical interaction between GFP−Vam7-Q284R and 3FLAG−Ykt6-R165Q was also detected. These results indicate that Ykt6 can form a complex with the vacuolar Q-SNAREs although this complex may be minor than the complex containing Nyv1. Since deletion of the *NYV1* gene did not increase the interaction between Vam7 and Ykt6 (Fig. S3), this pairing may be occurring constitutively, albeit at a low level.

### Ykt6 can function as a backup for plasma membrane R-SNAREs

Sec22 is an R-SNARE involved in vesicular transport between the endoplasmic reticulum (ER) and the Golgi complex (40). Unlike other ER/Golgi SNAREs, the deletion of the *SEC22* gene is not lethal. Biochemical and genetic analyses indicated that Ykt6 could substitute for Sec22 in ER-Golgi transport. Therefore, we examined whether *ykt6-102* and *ykt6-104* mutations exhibited genetic interactions with the *sec22Δ* mutation. As expected, deletion of the *SEC22* gene impaired the growth of the *ykt6-102* and *ykt6-104* strains (Fig. S4), confirming that Sec22 and Ykt6 functionally overlap in ER-Golgi transport. This suggests that the *ykt6-102* and *ykt6-104* mutations affected general SNARE function rather than having a defect in a specific association with vacuole SNAREs.

**Figure 9:**
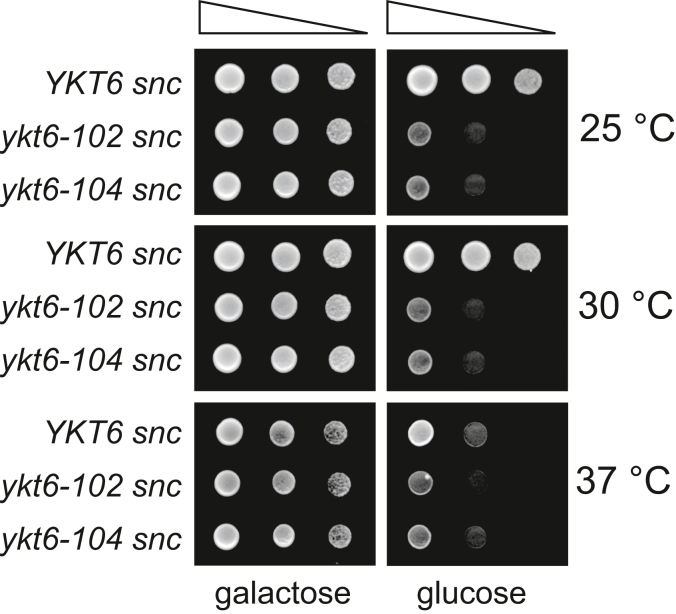
**Genetic interaction between *ykt6* and *snc* mutations.** The *ykt6Δ snc2Δ P*_*gal1*_*-SNC1* cells harboring pRS413-Ykt6 (YHW504), Ykt6-102 (YHW505), or Ykt6-104 (YHW506) were grown in SCGal medium to an early log phase at 25 °C. The cells equivalent to 10^−2^ to 10^−4^*A*_600_ units were spotted onto SCGal (galactose) and SC (glucose) agar media and incubated for 3 days at indicated temperatures. SC, synthetic complete.

Given the wide range of redundancies in Ykt6, we considered whether Ykt6 could be an alternative to the other R-SNAREs, Snc1 and its paralog Snc2. Snc1/2 forms an exocytic SNARE complex with Sso1/2 (Qa-SNARE) and Sec9 (Qbc-SNARE) to fuse Golgi-derived secretory vesicles with the plasma membrane (6). To test this hypothesis, we performed genetic interaction analysis between *SNC1/2* and *YKT6* using the *P*_*gal1*_*-SNC1 snc2Δ* (hereafter referred to as *snc*) strain, in which Snc proteins can be depleted by incubating in a glucose-containing medium. The *snc, ykt6-102 snc*, and *ykt6-104 snc* strains were all able to grow on galactose medium at any temperature tested (Fig. 9, galactose). Upon depletion of Snc proteins on glucose medium, the *ykt6-102 snc* and *ykt6-104 snc* strains were severely affected in growth at all temperatures, while the *snc* strain was viable below 30 °C, as shown previously (Fig. 9, glucose) (41). These results suggest that Ykt6 may complement the role of Snc proteins when they are depleted.

**Figure 10:**
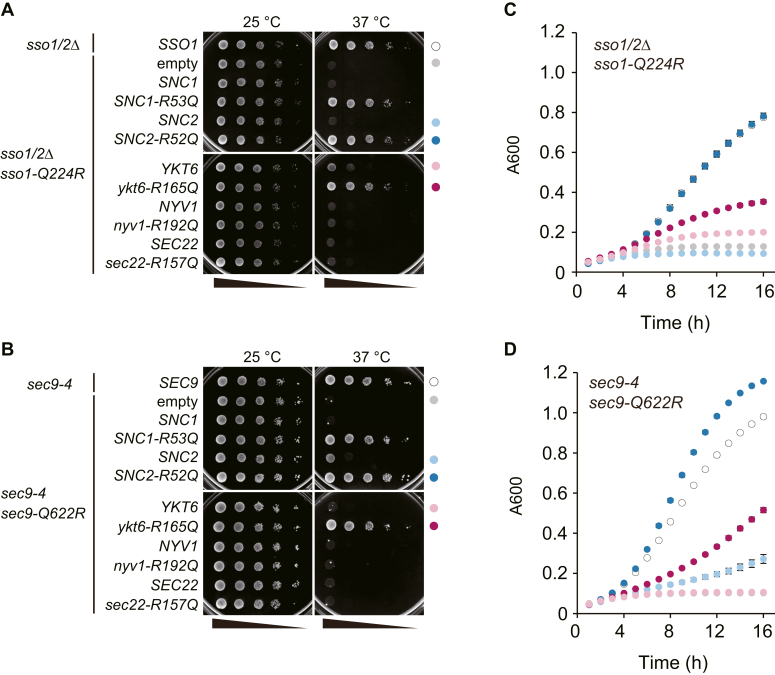
**Ykt6 interacts with the exocytic Q-SNAREs Sso1 and Sec9.***A*, the *P*_*gal1*_*-SSO1 sso2Δ* strain (YHW134) containing the plasmid encoding Sso1-Q224R was transformed with plasmids encoding WT R-SNARE (Snc1, Snc2, Ykt6, Nyv1, or Sec22), plasmids encoding mutant R-SNARE in which the 0-layer amino acid residue was replaced from R to Q (Snc1-R53Q, Snc2-R52Q, Ykt6-R165Q, Nyv1-R192Q, or Sec22-R157Q), or an empty vector. The cells were grown in SC−Ura/His medium to the early log phase at 25 °C. Next, cells equivalent to 10^−2^ to 10^−6^*A*_600_ units were spotted on SC−Ura/His medium plates and incubated for 3 days at 25 °C and 37 °C. The *P*_*gal1*_*-SSO1 sso2Δ* strain (YHW134) expressing WT Sso1 was used as a positive control. *B*, the *sec9-4* temperature-sensitive strain harboring the plasmid encoding Sec9-Q622R was transformed with either plasmids encoding WT R-SNARE (Snc1, Snc2, Ykt6, Nyv1, or Sec22), plasmids encoding mutant R-SNARE (Snc1-R53Q, Snc2-R52Q, Ykt6-R165Q, Nyv1-R192Q, or Sec22-R157Q), or an empty vector. The cells were grown in SC−His/Leu medium to the early log phase at 25 °C. Next, cells equivalent to 10^−2^ to 10^−6^*A*_600_ units were spotted on SC−His/Leu medium plates and incubated for 3 days at 25 °C and 37 °C. The *sec9-4* strain transformed with the plasmid encoding WT Sec9 was used as a positive control. *C*, the *P*_*gal1*_*-SSO1 sso2Δ* strain (YHW134) containing the plasmid encoding Sso1-Q224R and either the plasmid encoding Snc2 (*light blue*), Snc2-R52Q (*blue*), Ykt6 (*pink*), Ykt6-R165Q (*magenta*), or an empty vector (*light gray*) was used for analysis. The strains were pregrown at 25 °C as in *A* and inoculated in the same medium to grow at 37 °C as shown in Experimental procedures. Absorbance at 600 nm was automatically measured on a BioTek EPOCH 2 microplate reader. The *P*_*gal1*_*-SSO1 sso2Δ* strain (YHW134) expressing WT Sso1 was used as a positive control (*open circle*). *D*, the *sec9-4* strain containing the plasmid encoding Sec9-Q622R and either the plasmid encoding Snc2 (*light blue*), Snc2-R52Q (*blue*), Ykt6 (*pink*), Ykt6-R165Q (*magenta*), or an empty vector (*light gray*) was used for analysis. The strains were pregrown at 25 °C as in (*B*) and analyzed as in (*C*). The *sec9-4* strain expressing WT Sec9 was used as a positive control (*open circle*). SC, synthetic complete; SNARE, soluble N-ethylmaleimide-sensitive factor attachment protein.

Next, to examine whether Snc1/2 is exchangeable with Ykt6, we conducted a complementary substitution assay using the *sso1Δsso2Δ* strain expressing WT Sso1 from the *GAL1* promoter and mutant Sso1-Q224R, whose 0-layer glutamine residue was substituted to arginine, from the *SSO1* promoter (referred to as *sso1-Q224R sso2Δ*) (35). The strain with a plasmid encoding WT or mutant R-SNARE, whose 0-layer arginine residue was substituted to glutamine, was grown in a liquid medium (SC−Ura/His medium) at 25 °C, spotted on a glucose-containing medium, and grown at 25 °C and 37 °C. The strain with the empty plasmid grew at 25 °C but not at 37 °C (Fig. 10*A*), confirming that Sso1-Q224R was temperature-sensitive (35) and that WT Sso1 was successfully depleted in a medium containing glucose. Introduction of mutant *SNC1* (*SNC1-R53Q*) or *SNC2* (*SNC2-R52Q*) alleles, but not WT *SNC1* or *SNC2*, restored cell growth at 37 °C (Fig. 10*A*), confirming that the exocytic SNARE complex in the 2Q:2R composition lost its function at the restrictive temperature and became functional when the 3Q:1R ratio was restored. Surprisingly, the expression of the R-to-Q mutant of Ykt6, but not the WT Ykt6, restored cell growth, whereas the R-to-Q mutants of other R-SNAREs, Sec22-R157Q, and Nyv1-R192Q, did not (Fig. 10*A*). To test the reproducibility of SNARE pairing between Ykt6 and exocytic Q-SNAREs, we used a Q-to-R substitution in another Q-SNARE, Sec9. In this study, we used a strain carrying a temperature-sensitive *sec9-4* allele encoding Sec9 with a G458D substitution as the host strain. The Sec9-4 protein is known to significantly lose its ability to interact with Sso1 and Snc2 (42). Herein, *sec9-4* cells transformed with plasmids containing *sec9-Q622R* and either the WT or R-to-Q mutated R-SNARE genes were spotted on SC−His/Leu plates and incubated at the permissive or restrictive temperature. As observed for Sso1-Q224R, the expression of Ykt6-R165Q, but not WT Ykt6, restored the growth defects at restriction temperature (Fig. 10*B*), suggesting that the loss of the 3Q:1R composition by Sec9-Q622R was restored by Ykt6-R165Q. Taken together, these results suggest that Snc1/2 may be exchangeable with Ykt6 in exocytic SNARE complexes.

To accurately analyze growth recovery by restoring the 3Q:1R combinations, the growth rate of transformants in liquid medium was observed at 37 °C. The *sso1-Q224R sso2Δ* strain with the *SNC2-R52Q* plasmid grew as fast as the *SSO1 sso2Δ* strain, suggesting that the pairing of Sso1-Q224R and Snc2-R52Q may be fully functional. On the other hand, the *ykt6-R165Q* plasmid partially restored growth of the *sso1-Q224R sso2Δ* strain, and the empty, WT *SNC2*, and WT *YKT6* plasmids failed to complement temperature-sensitive growth of the *sso1-Q224R sso2Δ* strain (Fig. 10*C*). Similarly, the expression of Ykt6-R165Q partially restored growth of the *sec9-Q622R* strain, while Snc2-R52Q completely restored it (Fig. 10*D*). These results suggest that the role of Ykt6 at the plasma membrane remains minor but is sufficient for survival in case exocytic R-SNAREs become dysfunctional.

## Discussion

The *nyv1Δ* strain has no defects in any vesicular transport pathway, even though Nyv1 has been thoroughly characterized *in vitro* as a SNARE protein required for homotypic vacuole fusion. In the present study, we showed that the R-SNAREs Nyv1 and Ykt6 functionally overlap in homotypic vacuole fusion *in vivo*. The newly identified *ykt6-104* mutant showed no apparent defects in vacuolar transport except for the fusion of autophagosomes with vacuoles. Importantly, the *ykt6-104 nyv1Δ* double mutant exhibits highly fragmented vacuoles. Given that Ykt6 has a longin domain with acyltransferase activity, which palmitoylates Vac8 required for homotypic vacuole fusion, in its N-terminal region (43), we cannot rule out the possibility that the *ykt6-104* mutation affected Vac8 acylation, thereby revealing the *nyv1Δ* phenotype. However, complementary 0-layer mutations showed that Nyv1 and Ykt6 interacted with vacuole SNAREs in a mutually exclusive manner (Fig. 7). Previous *in vitro* analyses suggested that Ykt6 is involved in homotypic vacuolar fusion. Ykt6 binds to the ternary complex of Vam3-Vti1-Vam7, but equimolar amounts of Nyv1 inhibit this interaction, suggesting that Ykt6 and Nyv1 compete for binding with vacuolar Q-SNAREs (10). In addition, vacuoles isolated from *nyv1Δ* cells can be homotypically fused in an Ykt6-dependent manner when excess recombinant Vam7 proteins are added to the *in vitro* fusion reaction (20). These results are consistent with our genetic results, suggesting that Nyv1 can be substituted with Ykt6 for homotypic vacuole fusion. Moreover, the intracellular concentration of Vam7 is likely high enough for Ykt6 to exert its function in *nyv1Δ* cells. Coimmunoprecipitation experiments also support the idea that Ykt6 fulfills its role in a vacuolar R-SNARE (Fig. 8). However, this interaction appeared to be less efficient than the interaction between Nyv1 and Vam7, suggesting that Nyv1 is the primary vacuolar R-SNARE and Ykt6 may act as a backup at the vacuole. Deletion of the *NYV1* gene did not alter the amount of Ykt6 that coprecipitated with Vam7 (Fig. S3), suggesting that the association of Ykt6 with vacuolar Q-SNAREs occurs constitutively rather than being induced in the absence of Nyv1.

We also found that the *ykt6-104* allele genetically interacted with the *sec22Δ*, *nyv1*Δ, and *snc* mutations. These results indicate that the function of Ykt6 partially overlaps with that of Sec22, Nyv1, and Snc1/2. Complementary 0-layer mutation experiments done by Graf *et al.* (38) and us also suggest that Ykt6 can be exchanged with Sec22, Nyv1, and Snc1/2 in the Golgi, vacuolar, and exocytic SNARE complexes, respectively. A liposome fusion assay revealed that artificial protein-anchored Ykt6 on liposomes is fusogenic with the exocytic Q-SNAREs Sso1 (Qa) and Sec9 (Qbc) anchored on liposomes (6). This supports the hypothesis that Ykt6 acts with the exocytic Q-SNAREs Sso1 and Sec9 *in vivo*. However, the role of Ykt6 as an exocytic R-SNARE appears to be limited, as Ykt6-R165Q could only partially complement the growth defects of the *sso1-Q224R sso2Δ* and *sec9-Q622R* (Fig. 10). This is consistent with the fact that the *snc1Δsnc2Δ* strain exhibits severe growth defects (41). Reflecting these results, no physical interaction between Sso1 and Ykt6 could be detected in our experimental condition (Fig. S5), suggesting that Ykt6 associates inefficiently with exocytic Q-SNAREs compared to Snc2. In contrast to Ykt6, the R-to-Q substitution of either Sec22 or Nyv1 did not restore the growth defect of the *sso1-Q224R sso2Δ* strain (Fig. 10), although Sec22 and Nyv1 were fusogenic with Sso1 and Sec9 in liposome fusion *in vitro* (6). Sec22 and Nyv1 have transmembrane domain at their C termini and primarily localize to the ER/Golgi and vacuoles, respectively (18, 44), and may thus be limited in functioning as exocytic SNAREs. Ykt6 cycles between the cytosol and membranes, which is governed by reversible lipidation at its C-terminal CAAX motif, where C is cysteine, the 2 A residues are aliphatic amino acids, and X is one of several amino acids (45, 46). The F42S mutation in Ykt6 causes a loss of cytosolic localization, thereby highlighting its localization in various organelle membranes, including the plasma membrane, in *S. cerevisiae* (46). It is unknown whether Ykt6 localizes to secretory vesicles; however, the broad localization of Ykt6 throughout the vesicular transport network suggests that Ykt6 interacts with multiple Q-SNARE complexes.

In this study, we provide the genetic evidence that Ykt6 functionally overlaps, albeit in part, with virtually all R-SNAREs in *S. cerevisiae*. Yeasts have a relatively simple endomembrane system compared to mammalian and plant cells. Thus, the SNARE set is simple for yeast (47, 48). In particular, there are fewer R-SNAREs than Qa-, Qb-, and Qc-SNAREs; therefore, the functional overlap between Ykt6 and other R-SNAREs may contribute to maintaining the robustness of vesicular trafficking in *S. cerevisiae*.

## Experimental procedures

### Yeast strains and growth conditions

The *S. cerevisiae* strains used in this study are listed in Table S1. Gene disruption, gene tagging, and promoter replacement were performed as described by Gueldener *et al.* (49) and Janke *et al.* (50). Briefly, the entire open reading frame was replaced with selection markers such as *kanMX4*, *natNT2*, and *Kluyveromyces lactis LEU2* using PCR primers containing 45 nucleotides identical to the regions flanking the ORF (Table S2). The *YKT6* gene was disrupted in a diploid strain of BY4743, and the resulting heterozygous strain was transformed with pAR5 (21), obtaining the *ykt6*Δ haploid strain harboring pAR5 (YSU1). A series of *ykt6* mutants were obtained *via* plasmid shuffling from YSU1. *3FLAG-YKT6* gene was expressed under the control of the *YKT6* promoter by integrating *Eco*RV-digested pRS303-3FLAG-Ykt6 into the *YKT6* promoter region of YSU1, YSU39, and YTS930 strains, followed by removing pAR5. Yeast cells were grown in YPD medium (1% yeast extract, 2% peptone, and 2% glucose), synthetic dextrose (SD) medium (0.67% yeast nitrogen base without amino acids, 2% glucose), synthetic complete (SC) medium (0.67% yeast nitrogen base without amino acids, 2% glucose, and 0.2% dropout mixture), or synthetic casamino acid dextrose (SCD) medium (0.67% yeast nitrogen base without amino acids, 0.5% casamino acids, 2% glucose, 0.002% adenine, 0.002% uracil, and 0.002% tryptophan). For induction of genes under control of the *GAL1* promoter, SCGal medium (0.67% yeast nitrogen base without amino acids, 2% galactose, and 0.2% dropout mixture) was used. For nitrogen starvation, an SD(−N) medium (0.17% yeast nitrogen base without ammonium sulfate and amino acids and 2% glucose) was used. For growth curves, yeasts were grown in a 96-well plate filled with 220 μl of SC medium and sealed with a gas permeable polyurethane membrane Breathe-EASY (Sigma-Aldrich) using BioTek Epoch 2 microplate reader (Agilent Technologies).

### Plasmid construction

The plasmids used in this study are listed in Table S3. Plasmids were constructed using a conventional cloning method with restriction endonucleases and T4 DNA ligase, the gap repair cloning method (51, 52), In-Fusion cloning using an In-Fusion Snap Assembly Master Mix (Takara Bio Inc), or a seamless cloning method described by Liu *et al.* (53). Oligonucleotide primers used for plasmid constructions were listed in Table S4. DNA fragments used for plasmid construction *via* gap repair cloning and seamless cloning were listed in Tables S5 and S6, respectively. The detailed methods for the preparation of individual plasmids are described in the Supporting Information.

### Analyses of protein processing

Whole-cell extracts were prepared as described by Huang *et al.* (22). For the GFP–Atg8 processing assay, yeast cells were grown in SCD−Ura medium at 23 °C to the early log phase. After 1 ml of the culture was harvested as nonstarved cells, the remaining culture medium was centrifuged to collect the cells. The resulting cells were washed with distilled water and resuspended in SD(−N) medium, followed by dividing into two test tubes and incubating at 23 °C and 37 °C, respectively, for 2 h. One milliliter of the culture was harvested and used to prepare the cell extracts. Samples equivalent to 0.1 *A*_600_ were subjected to Western blot analysis and probed with an anti-GFP antibody (mFX75, FUJIFILM Wako Pure Chemical Corporation). For the processing of alkaline phosphatase (Pho8) and CPY (Prc1), cells were grown in YPD medium to the early log phase at 30 °C or 37 °C, the whole-cell extracts were collected, and subjected to Western blot analysis probed with mouse monoclonal anti-Pho8 (ab113688, Abcam plc) and anti-Pgk1 (ab113687, Abcam plc) antibodies and rabbit polyclonal anti-Prc1 antiserum (54). Immunoreactive protein bands were detected with horseradish peroxidase-conjugated goat anti-mouse IgG (Bio-Rad Laboratories) or anti-rabbit IgG (Bio-Rad) and chemiluminescence with Chemi-Lumi One L (Nacalai Tesque). Signal detection was performed using the ImageQuant LAS-4000 image analyzer (Cytiva), and the amounts of protein were quantified with ImageQuant TL software (Cytiva).

### GFP–Atg8 protection assay

Cells were grown to the early log phase in SCD at 23 °C then starved in SD(−N) medium at 37 °C for 3 h. The GFP–Atg8 protection assay was performed as described by Nair *et al.* (27).

### Colony immunoblotting

Colony immunoblotting was performed as described previously (55) with slight modifications. After yeast cells were grown to the early log phase in YPD medium at 23 °C and washed with sterile water, 4 *A*_600_ units of the cells were spotted on nitrocellulose membranes placed on an SD agar medium supplemented with 0.002% uracil, 0.01% leucine, 0.002% histidine, and 0.002% lysine and incubated at 37 °C for 18 to 20 h. The membranes were then removed from the agar medium, washed twice with deionized water, and immunoblotted with anti-Prc1 and anti-Pgk1 antibodies.

### Fluorescence microscopy

Cells expressing GFP-tagged proteins were grown in SCD medium to the early log phase and further incubated in SCD medium containing 0.2 μg/ml rapamycin for 2 h if necessary. The vacuolar membrane and vacuole lumen were labeled with ViVidFluor Neuro Red (CAS 162112-35-8, FUJIFILM Wako Pure Chemical Corporation), which is equivalent to FM 4-64, and CellHunt Blue CMAC (CAS 147963-22-2, Setareh Biotech), respectively, as described by Conibear and Stevens (56). For fluorescence microscopy analysis, cells were visualized under a 100× oil immersion objective (1.40 NA; Olympus) using an Olympus IX71 fluorescence microscope equipped with a Zyla 4.2 PLUS sCMOS camera (Andor Technology) and processed using MetaMorph version 7.10 imaging software (Molecular Devices).

### Protein extraction and coimmunoprecipitation

Cells expressing GFP- and FLAG-tagged SNARE proteins were grown in 100 ml of SC−Ura/His medium to the midlog phase and 80 *A*_600_ units of the cells were collected by centrifugation at 1200*g* for 5 min. The cells were resuspended in 10 ml of spheroplast buffer [1 M sorbitol, 20 mM Hepes-NaOH (pH 7.4)] containing 1 mg of Zymolyase-100T (Nacalai Tesque), and incubated for 30 min at 30 °C with shaking at 160 rpm. Spheroplasts were collected by centrifugation and resuspended in 10 ml of spheroplast buffer containing 2 mg of dithiobis (succinimidyl propionate) (Dojindo Laboratories), and incubated for 30 min at 30 °C with shaking at 100 rpm. To quench the cross-linking, Tris–HCl (pH 7.6) was added to a final concentration of 0.1 M and were incubated for 15 min at 30 °C with shaking at 100 rpm. Spheroplasts were collected by centrifugation at 1200*g* for 10 min and resuspended in 0.9 ml of lysis buffer [20 mM Pipes-NaOH (pH 6.8), 200 mM sorbitol, 150 mM NaCl, 5 mM MgCl_2_, 1 mM PMSF, 1×cOmplete protease inhibitor cocktail (Sigma-Aldrich)], and 0.1 ml of 10% Triton X-100 was added. After standing on ice for 10 min, the supernatant was collected as the total protein fraction by centrifugation at 500*g* for 10 min. For immunoprecipitation, 10 μl of GFP-Trap magnetic agarose beads (Proteintech Group) were washed with 500 μl of lysis buffer using MagRack 6 (Cytiva), and then 800 μl of the total protein fraction was added. After incubation for 2 h at 4 °C with rotation, the supernatant was removed using the magnetic rack. The beads were then washed three times with 500 μl of lysis buffer using the magnetic rack and subsequently resuspended in 200 μl of SDS sample buffer [62.5 mM Tris–HCl (pH 6.8), 2% SDS, 10% glycerol, 5% β-mercaptoethanol, and bromophenol blue]. The suspension was boiled for 5 min at 95 °C, and the supernatant was used for SDS-PAGE and Western blot analysis with anti-GFP antibody (7.1 and 13.1, Sigma-Aldrich), and mouse monoclonal anti-DYKDDDDK tag antibody (1E6, FUJIFILM Wako Pure Chemical Corporation).

## Data availability

All data are in the manuscript.

## Supporting information

This article contains supporting information (21, 35, 53, 57, 58, 59, 60, 61, 62, 63).

## Conflict of interest

The authors declare that they have no conflicts of interest with the contents of this article.
