# Supplementary material for: Ykt6 functionally overlaps with vacuolar and exocytic R-SNAREs in the yeast Saccharomyces cerevisiae
Source: J Biol Chem. 2024 Apr 6;300(5):107274. doi: 10.1016/j.jbc.2024.107274 (PMC11091695; doi:10.1016/j.jbc.2024.107274)
Supplement: Supporting Information [file mmc1.pdf]

Ykt6 functionally overlaps with vacuolar and exocytic R-SNAREs in the yeast *Saccharomyces cerevisiae*

Hayate Watanabe, Shingo Urano, Nozomi Kikuchi, Yurika Kubo, Ayumi Kikuchi, Katsuya Gomi, and Takahiro Shintani

Supporting Information

Plasmid construction

The oligonucleotides used for plasmid construction are shown in Table S4.

Ykt6 plasmids

The promoter and terminator regions of *YKT6* were PCR-amplified with combinations of the oligonucleotides yp1 and yp2, and yp3 and yp4, respectively. Yeast cells were co-transformed with these fragments and *Sac*I/*Kpn*I-digested pRS416 to assemble them into a circular plasmid, termed pRS416-YKT6prom/term. *Sal*I-digested pRS416-YKT6prom/term and the *Sac*I/*Eco*RI fragment of *ykt6-13* from pYK11 (a gift from Dr. Stevens) were then assembled in yeast cells to generate pRS416-Ykt6-13. The *Sac*I/*Xho*I fragment of *3FLAG-YKT6* from pRS416-3FLAG-Ykt6 was subcloned to pRS413 to generate pRS413-3FLAG-Ykt6. Plasmids containing the *YKT6* gene or its derivatives were constructed via the gap repair cloning method using PCR products amplified with the primers and DNA templates shown in Table S5. The plasmid pRS413-3FLAG-Ykt6-R165Q was generated by seamless cloning (1) using PCR products and vector DNAs, as shown in Table S6. To construct pRS423-Vam3-Vti1-3FLAG-Ykt6, the *VAM3−VTI1* fragment was amplified by fusion PCR using the oligonucleotides oTAKA551, oTAKA552, oTAKA553, and oTAKA554, and the resulted fragment was assembled with pRS423-3FLAG-Ykt6 digested with *Sac*I. The 3FLAG-Ykt6-R165Q fragment was obtained by the digestion of pRS423-3FLAG-Ykt6-R165Q with *Bam*HI and *Cla*I and was ligated to pRS423-Vam3-Vti1-3FLAG-Ykt6 digested with *Bam*HI and *Cla*I to generate pRS423-Vam3-Vti1-3FLAG-Ykt6-R165Q. The *SEC9* fragment was PCR-amplified with oligonucleotides oTAKA555 and oTAKA556, and then assembled into *Sac*I-digested pRS423-3FLAG-Ykt6 and pRS423-3FLAG-Ykt6-R165Q via In-Fusion cloning to produce pRS423-Sec9-3FLAG-Ykt6 and pRS423-Sec9-3FLAG-Ykt6-R165Q, respectively.

Vam7 plasmids

The plasmids pRS415-Vam7, pRS415-Vam7-Q284R, pCu416-GFP-Vam7, and pCu416-GFP-Vam7-Q284R were generated via the gap repair cloning method using PCR products and vector DNAs shown in Table S5.

Nyv1 plasmids

NYV1 was PCR-amplified with the oligonucleotides AK3 and AK4, followed by digestion with *Bam*HI and *Eag*I to ligate pRS413 to generate pRS413-Nyv1. The plasmid pRS413-Nyv1-R192Q was generated by gap repair cloning using PCR products and vector DNAs, as shown in Table S5. The plasmids pRS413-3FLAG-Nyv1 and pRS413-3FLAG-Nyv1-R192Q were generated by seamless cloning using PCR products and vector DNAs, as shown in Table S6.

Sec22 plasmids

Plasmids pRS416-Sec22 and pRS416-Sec22-R157Q were generated via the gap repair cloning method using PCR products and vector DNAs, as shown in Table S5. These plasmids were digested with *Xba*I/*Xho*I, and the resulting Sec22 and Sec22-R157Q fragments were ligated into pRS413 to generate pRS413-Sec22 and pRS413-Sec22-R157Q, respectively.

Snc1 plasmids

The *SNC1* gene fragment was PCR-amplified with oligonucleotides AK25 and AK26 and digested with *Bam*HI and *Hind*III. The resulting *Bam*HI/*Hind*III fragment was ligated into the pRS425 vector to generate pRS425-Snc1. The *Sac*I/*Sal*I fragment of pRS425-Snc1 was subcloned into pRS413 to generate pRS413-Snc1. pRS413-Snc1-R53Q was constructed via the gap repair cloning method using PCR products amplified with the primers and DNA templates listed in Table S5.

Snc2 plasmids

The *SNC2* gene was PCR-amplified using oHW37 and oHW38 and assembled into *Bam*HI/*Sac*I-digested pRS413 plasmids via In-Fusion cloning to produce pRS413-Snc2. The *SNC2-R52Q* fragment was excised from pRS316-Snc2-R52Q (a gift from Dr. Brennwald) and ligated into pRS413 to generate pRS413-Snc2-R52Q. The plasmids pRS413-3FLAG-Snc2 and pRS413-3FLAG-Snc2-R52Q were generated by seamless cloning using PCR products and vector DNAs, as shown in Table S6.

Sso1 plasmids

Plasmids pRS316-Sso1 and pRS316-Sso1-Q224R were a gift from Dr. Brennwald. The *GAL1* promoter-*CYC1* terminator fragment was excised from p416GAL (2) with *Sac*I and *Kpn*I and then ligated to pRG205 (Addgene) (3) to generate pRG205GAL. The *SSO1* gene was PCR-amplified with the primers oHW29 and oHW30, and then assembled into *Bam*HI/*Hin*dIII-digested pRG205GAL plasmids via In-Fusion cloning to produce pRG205GAL-Sso1. The plasmids pCu416-GFP-Sso1 and pCu416-GFP-Sso1-Q224R were generated by gap repair cloning using PCR products and vector DNAs, as shown in Table S5.

Sec9 plasmids

The *SEC9* gene was PCR-amplified using oHW42 and oHW43 and assembled into *Bam*HI/*Sal*I-digested pRS315 plasmids via In-Fusion cloning to produce pRS315-Sec9. The plasmid pRS315-Sec9-Q622R was a gift from Dr. Brennwald.

Plasmids for GFP-tagged proteins

The oligonucleotides oTAKA303 and oTAKA304 were used as primers to PCR-amplify yeGFP DNA fragments using pYM25 (4) as template DNA, which was digested with *Bam*HI and ligated to *Bam*HI-digested pRS316GFP-AUT7 plasmids (5) to generate pRS316-yeGFP-Atg8. DNA fragments containing the *PHO8* coding sequence and its terminator region were PCR-amplified using the oligonucleotides oTAKA491 and oTAKA492 and assembled into *Eco*RI/*Kpn*I-digested pCuGFP(416) plasmids (6) via In-Fusion cloning to generate pCu416-GFP-Pho8. To generate pCu306-GFP-Pho8, pCu416-GFP-Pho8 was digested with *Eco*RI/*Kpn*I and the resulting *Eco*RI/*Kpn*I *PHO8* fragment was ligated into pCuGFP(306). DNA fragments containing the *VPH1*-coding sequence and its promoter region were PCR-amplified using the primers oTAKA497 and oTAKA498 and assembled into *Not*I/*Spe*I-digested p316-GFP-ADHt plasmids (7) via In-Fusion cloning to produce pRS316-Vph1-GFP. To generate pRS306-Vph1-GFP, pRS316-Vph1-GFP was digested with *Sac*I/*Kpn*I, and the resulting *Sac*I/*Kpn*I fragment of *VPH1-GFP* was subcloned into the plasmid pRS306.

Table S1. Yeast strains used in this study.

Strain name	Genotype	Reference or source	Experiments used
BY4742	*MATα ura3Δ0, leu2Δ0, his3Δ1, lys2Δ0*	(8)	Strain construction
			Figs. 8, S3, and S5
YSU1	BY4742; *ykt6Δ::kanMX4* [pAR5]	This study	Strain construction, Fig. S4
YSU34	BY4742; *ykt6Δ::kanMX4* [pRS413-Ykt6]	This study	Figs. 1−6
YSU37	BY4742; *ykt6Δ::kanMX4* [pRS413-Ykt6-13]	This study	Figs. 1−5
YTS856	BY4742; *ykt6Δ::kanMX4* [pRS413-Ykt6-101]	This study	Fig. 1
YTS858	BY4742; *ykt6Δ::kanMX4* [pRS413-Ykt6-102]	This study	Figs. 1−6
YTS860	BY4742; *ykt6Δ::kanMX4* [pRS413-Ykt6-103]	This study	Fig. 1
YTS862	BY4742; *ykt6Δ::kanMX4* [pRS413-Ykt6-104]	This study	Figs. 1−6
YTS864	BY4742; *ykt6Δ::kanMX4* [pRS413-Ykt6-105]	This study	Fig. 1
YTS866	BY4742; *ykt6Δ::kanMX4* [pRS413-Ykt6-106]	This study	Fig. 1
YTS837	BY4742; *ykt6Δ::kanMX4 atg2Δ::LEU2Kl* [pRS413-YKT6]	This study	Fig. 2
YTS854	BY4742; *ykt6Δ::kanMX4 atg2Δ::LEU2Kl* [pRS413-Ykt6-102]	This study	Fig. 2
YTS843	BY4742; *ykt6Δ::kanMX4 atg2Δ::LEU2Kl* [pRS413-Ykt6-104]	This study	Fig. 2
vam3Δ	BY4742; *vam3Δ::kanMX4*	Open Biosystems	Fig. 2
apl5Δ	BY4742; *apl5Δ::kanMX4*	Open Biosystems	Fig. 4
YHW221	BY4742; *PHO8::*P_{cup1}*−GFP−PHO8 URA3 ykt6Δ::kanMX4* [pRS413-Ykt6]	This study	Fig. 4
YHW233	BY4742; *PHO8::*P_{cup1}*−GFP−PHO8 URA3 ykt6Δ::kanMX4* [pRS413-Ykt6-13]	This study	Fig. 4
YHW223	BY4742; *PHO8::*P_{cup1}*−GFP−PHO8 URA3 ykt6Δ::kanMX4* [pRS413-Ykt6-102]	This study	Fig. 4
YHW225	BY4742; *PHO8::*P_{cup1}*−GFP−PHO8 URA3 ykt6Δ::kanMX4* [pRS413-Ykt6-104]	This study	Fig. 4
YHW491	BY4742; *PHO8::*P_{cup1}*−GFP−PHO8 URA3 apl5Δ::kanMX4*	This study	Fig. 4
YHW581	BY4742; *PHO8::*P_{cup1}*−GFP−PHO8 URA3 ykt6Δ::kanMX4 SEC7−mCherry::LEU2Kl* [pRS413-Ykt6]	This study	Fig. 4

Table S1. Continued

Strain name	Genotype	Reference or source	Experiments used
YHW582	BY4742; *PHO8::P_{cup1}−GFP−PHO8 URA3 ykt6Δ::kanMX4 SEC7−mCherry::LEU2Kl* [pRS413-Ykt6-104]	This study	Fig. 4
YHW585	BY4742; *PHO8::P_{cup1}−GFP−PHO8 URA3 ykt6Δ::kanMX4 SEC7−mCherry::LEU2Kl apl5Δ::kanMX4*	This study	Fig. 4
YHW227	BY4742; *VPH1::VPH1−GFP URA3 ykt6Δ::kanMX4* [pRS413-Ykt6]	This study	Fig. 5
YHW235	BY4742; *VPH1::VPH1−GFP URA3 ykt6Δ::kanMX4* [pRS413-Ykt6-13]	This study	Fig. 5
YHW229	BY4742; *VPH1::VPH1−GFP URA3 ykt6Δ::kanMX4* [pRS413-Ykt6-102]	This study	Fig. 5
YHW231	BY4742; *VPH1::VPH1−GFP URA3 ykt6Δ::kanMX4* [pRS413-Ykt6-104]	This study	Fig. 5
vps16Δ	BY4742; *vps16Δ::kanMX4*	Open Biosystems	Fig. 5
YSU39	BY4742: *nyv1Δ::natNT2 ykt6Δ::kanMX4* [pAR5]	This study	Strain construction
YTS868	BY4742; *nyv1Δ::natNT2 ykt6Δ::kanMX4* [pRS413-Ykt6]	This study	Fig. 6
YTS872	BY4742; *nyv1Δ::natNT2 ykt6Δ::kanMX4* [pRS413-Ykt6-102]	This study	Fig. 6
YTS876	BY4742; *nyv1Δ::natNT2 ykt6Δ::kanMX4* [pRS413-Ykt6-104]	This study	Fig. 6
vam7Δ	BY4742; *vam7Δ::kanMX4*	Open Biosystems	Figs. 7 and S1
YTS820	BY4742; *vam7Δ::kanMX4 ATG8::P_{atg8}−yeGFP−ATG8 URA3*	This study	Fig. S2
nyv1Δ	BY4742; *nyv1Δ::kanMX4*	Open Biosystems	Fig. S3
YTS930	BY4742; *sec22Δ::LEU2Kl ykt6Δ::kanMX4* [pAR5]	This study	Fig. S4
YHW504	BY4742: *snc1::natNT2−P_{gal1}−SNC1 snc2Δ::kanMX4 ykt6Δ::loxP* [pRS413-Ykt6]	This study	Fig. 9
YHW505	BY4742: *snc1::natNT2−P_{gal1}−SNC1 snc2Δ::kanMX4 ykt6Δ::loxP* [pRS413-Ykt6-102]	This study	Fig. 9
YHW506	BY4742: *snc1::natNT2−P_{gal1}−SNC1 snc2Δ::kanMX4 ykt6Δ::loxP* [pRS413-Ykt6-104]	This study	Fig. 9
YHW134	BY4742; *sso1Δ::kanMX4 sso2Δ::kanMX4 LEU2::P_{gal1}−SSO1*	This study	Fig. 10
sec9-4	BY4741; *sec9-4::kanMX4*	Euroscarf	Fig. 10

Table S2. Oligonucleotides and template DNA sequences used for gene disruptions, gene tagging, and promoter replacement in this study.

Target gene	Oligonucleotide sequences (5´> 3´)	Template DNA
YKT6 disruption	GGCATTTCTGAAGCTTTACGTATCACTAGAGAAGTTATTTTGGCAcagctgaagcttcgtacgc<br>GCAGATGAAAAGCCACACTATGCTGAAAATAGTGTGTGGATGCATcataggccactagtggatctga	pUG6
ATG2 disruption	TAAAGCAAATTAAGAGGAACCCTTTTTTTTTTTGATTTCGATACAcagctgaagcttcgtacgc<br>ATAAAATATGAATTGAATATATATCAAAAATGTCTGCAAAAATTTcataggccactagtggatctg	pUG73
NYV1 disruption	AATTTATTAAGCTGTTAGAGCATTGGACTTTTATATTTTTACCAAcagctgaagcttcgtacgc<br>ATTAATGTTATTGTCGTGGGACAGCTCCCCTTTTTTTTTATTTACcataggccactagtggatctga	pFA6a-natNT2
SEC22 disruption	AACCCTGACAGTGACACCCCGTTACACACTCACAATTAAGTAGGGcagctgaagcttcgtacgc<br>GACCAAATTGATCGGGATTTGTGATGTGGGATGATGGGGTGACGTcataggccactagtggatctga	pUG73
SNC2 disruption	CAGGCATACATTCGAAACACTTCCAAATACAAAATAAGAACGCGCcagctgaagcttcgtacgc<br>TGACTACTGTATATATTTTTTAGAATTAGCATCGGGAACCGATGAgcataggccactagtggatctga	pFA6a-natNT2
SEC7 tagging	AAACAATTTCTAAGCAGAGTTGGTGAATTATACCTTTCTACTGATgctgaagcttcgtacgctgc<br>aaacgtcgacatattaccctgttatccctagcgg	mCherry－V5
	agggtaatatgtcgacgtttgcaggtcgacaacccttaatat<br>CAACTAAGCATATTTTAATCTGCTGGACCATTCAACAAAGCCTTAgcataggccactagtggatctga	pUG73
SNC1 promoter	TAAAACTAGTCTAAATCCTCAACCTTCAGCTTCCAATCACTCCTTcagctgaagcttcgtacgc<br>gcataggccactagtggatctg	pFA6a-natNT2
	cagatccactagtggcctatgccggattagaagccgccgagc<br>GTGCTCGGATAGAGCATAAGGGTCAAAGGGAGTAGATGACGACATgatccactagttctagaatc	pYM-N22

Capital letters in the oligonucleotide sequence indicate the sequences homologous to the target genes.

Table S3. Plasmids used in this study.

Plasmid name	Genotype	Reference
pRS413	*CEN6/ARS HIS3*	(9)
pRS415	*CEN6/ARS LEU2*	(9)
pAR5	*CEN6/ARS URA3* P_{ykt6}*−YKT6*	(10)
pRS416-Ykt6-13	*CEN6/ARS URA3* P_{ykt6}*−ykt6-13* (K143N, E160G, Q164R, F186L, K188M)	This study
pRS413-Ykt6	*CEN6/ARS HIS3* P_{ykt6}*−YKT6*	This study
pRS413-Ykt6-13	*CEN6/ARS HIS3* P_{ykt6}*−ykt6-13* (K143N, E160G, Q164R, F186L, K188M)	This study
pRS413-Ykt6-101	*CEN6/ARS HIS3* P_{ykt6}*−ykt6-101* (E160G, Q164R, F186L, K188M)	This study
pRS413-Ykt6-102	*CEN6/ARS HIS3* P_{ykt6}*−ykt6-102* (K143N, F186L, K188M)	This study
pRS413-Ykt6-103	*CEN6/ARS HIS3* P_{ykt6}*−YKT6-103* (K143N, E160G, Q164R)	This study
pRS413-Ykt6-104	*CEN6/ARS HIS3* P_{ykt6}*−ykt6-104* (F186L, K188M)	This study
pRS413-Ykt6-105	*CEN6/ARS HIS3* P_{ykt6}*−YKT6-105* (E160G, Q164R)	This study
pRS413-Ykt6-106	*CEN6/ARS HIS3* P_{ykt6}*−YKT6-106* (K143N)	This study
pRS316-yeGFP-Atg8	*CEN6/ARS URA3* P_{atg8}*−yeGFP−ATG8*	This study
pCu306-GFP-Pho8	*CEN6/ARS URA3* P_{cup1}*−GFP−PHO8*	This study
pRS306-Vph1-GFP	*CEN6/ARS URA3* P_{vph1}*−VPH1−GFP*	This study
pRS415-Vam7	*CEN6/ARS LEU2* P_{vam7}*−VAM7*	This study
pRS415-Vam7-Q284R	*CEN6/ARS LEU2* P_{vam7}*−vam7-Q284R*	This study
pRS413-Ykt6-R165Q	*CEN6/ARS HIS3* P_{ykt6}*−ykt6-R165Q*	This study
pRS413-Nyv1	*CEN6/ARS HIS3* P_{nyv1}*−NYV1*	This study
pRS413-Nyv1-R192Q	*CEN6/ARS HIS3* P_{nyv1}*−nyv1-R192Q*	This study
pRS316-Snc2	*CEN6/ARS URA3* P_{snc2}*−SNC2*	(11)
pRS316-Snc2-R52Q	*CEN6/ARS URA3* P_{snc2}*−SNC2-R52Q*	(11)
pRS316-Sso1	*CEN6/ARS URA3* P_{sso1}*−SSO1*	(11)

Table S3. Continued

Plasmid name	Genotype	Reference
pRS316-Sso1-Q224R	*CEN6/ARS URA3* P_{sso1}*−sso1-Q224R*	(11)
pRS413-Snc1	*CEN6/ARS HIS3* P_{snc1}*−SNC1*	This study
pRS413-Snc1-R53Q	*CEN6/ARS HIS3* P_{snc1}*−SNC1-R53Q*	This study
pRS413-Snc2	*CEN6/ARS HIS3* P_{snc2}*−SNC2*	This study
pRS413-Snc2-R52Q	*CEN6/ARS HIS3* P_{snc2}*−SNC2-R52Q*	This study
pRS413-Sec22	*CEN6/ARS HIS3* P_{sec22}*−SEC22*	This study
pRS413-Sec22-R157Q	*CEN6/ARS HIS3* P_{sec22}*−sec22-R157Q*	This study
pRG205GAL-Sso1	*LEU2* P_{gal1}*−SSO1*	This study
pRS315-Sec9	*CEN6/ARS LEU2* P_{sec9}*−SEC9*	This study
pRS315-Sec9-Q622R	*CEN6/ARS LEU2* P_{sec9}*−sec9-Q622R*	(11)
pRS413-3FLAG-Ykt6	*CEN6/ARS HIS3* P_{ykt6}*−3FLAG-YKT6*	This study
pRS413-3FLAG-Ykt6-R165Q	*CEN6/ARS HIS3* P_{ykt6}*−3FLAG-ykt6-R165Q*	This study
pRS413-3FLAG-Nyv1	*CEN6/ARS HIS3* P_{nyv1}*−3FLAG-NYV1*	This study
pRS413-3FLAG-Nyv1-R192Q	*CEN6/ARS HIS3* P_{nyv1}*−3FLAG-nyv1-R192Q*	This study
pRS413-3FLAG-Snc2	*CEN6/ARS HIS3* P_{snc2}*−3FLAG-SNC2*	This study
pRS413-3FLAG-Snc2-R52Q	*CEN6/ARS HIS3* P_{snc2}*−3FLAG-SNC2-R52Q*	This study
pCu416-GFP-Vam7	*CEN6/ARS URA3* P_{cup1}*−GFP-VAM7*	This study
pCu416-GFP-Vam7-Q284R	*CEN6/ARS URA3* P_{cup1}*−GFP-vam7-Q284R*	This study
pCu416-GFP-Sso1	*CEN6/ARS URA3* P_{cup1}*−GFP-SSO1*	This study
pCu416-GFP-Sso1-Q224R	*CEN6/ARS URA3* P_{cup1}*−GFP-sso1-Q224R*	This study
pRS423-3FLAG-Ykt6	*2µ HIS3* P_{ykt6}*−3FLAG−YKT6*	This study
pRS423-3FLAG-Ykt6-R165Q	*2µ HIS3* P_{ykt6}*−3FLAG−ykt6-R165Q*	This study
pRS423-Vam3-Vti1-3FLAG-Ykt6	*2µ HIS3* P_{vam3}*−VAM3* P_{vti1}*−VTI1* P_{ykt6}*−3FLAG−YKT6*	This study

Table S3. Continued

Plasmid name	Genotype	Reference
pRS423-Vam3-Vti1-3FLAG-Ykt6-R165Q	*2μ HIS3* P_{vam3}*−VAM3* P_{vti1}*−VTI1* P_{ykt6}*−3FLAG−ykt6-R165Q*	This study
pRS423-Sec9-3FLAG-Ykt6	*2μ HIS3* P_{sec9}*−SEC9* P_{ykt6}*−3FLAG−YKT6*	This study
pRS423-Sec9-3FLAG-Ykt6-R165Q	*2μ HIS3* P_{sec9}*−SEC9* P_{ykt6}*−3FLAG−ykt6-R165Q*	This study

Table S4. Oligonucleotide primers used for plasmid constructions.

Primer name	Nucleotide sequence (5´>3´)
yp1	CGCAATTAACCCTCACTAAAGGGAACAAAAGCTGGAGCTCAAAGAGGCTTCCTATTAGGA
yp2	AGTGTGTGGATGCATCTGCAGAGAAGAAGTCGACTGCCAAAATAACTTC
yp3	GAAGTTATTTTGGCAGTCGACTTCTTCTCTGCAGATGCATCCACACACT
yp4	CTCACTATAGGGCGAATTGGGTACCGGGCCCCCCCTCGAGGGCCAAGTTGGTTAAGGCGT
M13 Fw	GTAAAACGACGGCCAGT
M13 Rv	CAGGAAACAGCTATGAC
oTAKA364	GACGCTATCATGAAAGTTCAACAAGAACTGGATG
oTAKA365	CTTGTTGAACTTTCATGATAGCGTCAGCCTGTG
oTAKA376	CGATTGAGAATGTTTTACAAAGAGGTGAAAAGTTGGATAATTTGG
oTAKA377	CACCTCTTTGTAAAACATTCTCAATCGTTTTGTGCAAAACG
oTAKA378	CCAAAATGTTTTATAAGCAAGCTAAAAAATCCAATTCG
oTAKA379	TTAGCTTGCTTATAAAACATTTTGGAACTTGCCG
oTAKA387	TATAAAGATCATGACATCGACTACAAGGATGACGATGACAAGGGATCCAGAATCTACTACATCGGTGTATTTCG
oTAKA388	TGTAGTCGATGTCATGATCTTTATAATCACCGTCATGGTCTTTGTAGTCCATTGCCAAAATAACTTCTCTAGTG
oSU43	GAATGTTTTACAACAAGGTGAAAAGTTGG
oSU44	CCAACTTTTCACCTTGTTGTAAAACATTC
oSU52	ATTCCTGCAGCCCGGGGTCGTGAGCAATGGCTAATAA
oSU53	CCGCTCTAGAACTAGTGCAACAGTACAATCTCAATG
oSU41	CAAACACGGAATGAGCTACTTAC
oSU42	TAGCTCATTCCGTGTTTGCAGC
AK3	TCGACGGATCCGGCGGCAAGACTTCAAGAAG
AK4	CAATTCGGCCGGACGTTCAGATTCTCCC

Table S4. Continued.

Primer name	Nucleotide sequence (5´>3´)
oSU37	AGTTCTTGGAGCAACAAGAAAGAGTTTC
oSU38	TTCTTGTTGCTCCAAGAACTTGTCG
AK31	CGAGGTCGACGGTATCGATGACACCACCCAACACCAAAAAC
AK32	CGAGAGAATCTCCCTGGTAAAGTAGGTCTTCGATGTTCTTGG
AK33	CCAAGAACATCGAAGACCTACTTTACCAGGGAGATTCTCTCG
AK34	CGCGGTGGCGGCCGCTCTAGAACTAGTAGGAATTATAACTTAATAC
AK25	GGTCGACGGTATCGATAAGCTTAAGTGATAAGATGAAATTAACGAGGGG
AK26	CTAGAACTAGTGGATCCGCTGGTTTGAACCAAGAATTGAAGACG
oHW21	TAAAGTAGCAGAACAAGGTGAAAGA
oHW22	AATCTTTCACCTTGTTCTGCTACTT
oHW37	GCAGCCCGGGGGATCGTGTATTATTAATACGAACAAAA
oHW38	GGGAACAAAAGCTGGGACGGCCACTAAAACTGAT
oHW29	TAGAACTAGTGGATCATGAGTTATAATAATCCGTACC
oHW30	CGGTATCGATAAGCTTTAACGCGTTTTGACAACGG
oHW42	TAGAACTAGTGGATCTCTCTCTCTCTCTCTACTTA
oHW43	CCCCCTCGAGGTCGAGCCCCCTGTTTTCGAAGG
oTAKA303	ATAAAAATAATTACTAGAGACATGGGATCCTCTAAAGGTGAAGAATTATTCACTGG
oTAKA304	ATATTCAGACTTAAATGTAGACTTGGATCCTTTGTACAATTCATCCATACCATGGG
oTAKA491	CGGGCTGCAGGAATTCATGATGACTCACACATTACCAAGCG
oTAKA492	TATAGGGCGAATTGGGTACCCGACGATGCCGATGATGAC
oTAKA497	ACCGCGGTGGCGGCCGCAAGAAGGATAAGGATGGGAAG
oTAKA498	CGGGGGATCCACTAGTGCTTGAAGCGGAAGAGCTTG

Table S4. Continued.

Primer name	Nucleotide sequence (5´>3´)
PNK034	GATCCGGCGGTGGTGGCTCTGGTGGAGGCGGTTCTATGGCAGCTAATTCTGTAGGG
PNK035	CGTGACATAACTAATTACATGACTCGAGTCAAGCACTGTTGTTAAAATG
oTAKA252	TGCAATATCATATAGAAGTCATCGACTAGTATGTCTAAAGGTGAAGAATTATTCAC
oTAKA254	CCACCAGAGCCACCACCGCCGGATCCACCGCCACCTTTGTACAATTCATCCATACC
MY170	ACATGGCATGGATGAACTATACAAGATGAGTTATAATAATCCGTACCAGTTGGAAACCCCTTTTG
MY171	AAACGACGGCCAGTGAGCGCGCGTATGCCCAACAAACCGTGCCTTAGTGAGATTTCGGTCGTAAC
oHW140	CTGGTAATAGAACGGCAAGAAAACG
oHW141	CGTTTTCTTGCCGTTCTATTACCAG
oHW98	TATAAAGATCATGACATCGACTACAAGGATGACGATGACAAGATGTCGTCATCAGTGCC
oHW99	GTCATGATCTTTATAATCACCGTCATGGTCTTTGTAGTCCATCGTTGCGCGTTCTTA
oHW100	TATAAAGATCATGACATCGACTACAAGGATGACGATGACAAGATGAAACGCTTTAATGGT
oHW101	GTCATGATCTTTATAATCACCGTCATGGTCTTTGTAGTCCATTTGGTAAAAATATAAAAG
oTAKA551	GGGAACAAAAGCTGGAGCTCCTGTGAGCTTGCGTTTACGG
oTAKA552	ATATAAGAGGAATTCGGGCATATCGAGCCCTTGC
oTAKA553	GATATGCCCGAATTCCTCTTATATATACACGGTCC
oTAKA554	TAGGAAGCCTCTTTGAGCTCAGGCAAGCTAAAAGATAAGG
oTAKA555	GGGAACAAAAGCTGGAGCTCTGGCGGTGGTTGTAGTTC
oTAKA556	TAGGAAGCCTCTTTGAGCTCGGTTAAGTTCATAAC

Table S5. Nucleic acids used for plasmid construction via *in vivo* assembly in yeast cells.

Plasmid name	PCR product used for *in vivo* assembly				Vector DNA used for *in vivo* assembly
		Forward primer	Reverse primer	Template DNA	
pRS416-YKT6prom/term	Fragment 1	yp1	yp2	genomic DNA	*Sac*I/*Kpn*I-digested pRS416
	Fragment 2	yp3	yp4	genomic DNA	
pRS413-Ykt6-13	Fragment 1	M13 Fw	M13 Rv	pRS416-Ykt6-13	*Bam*HI/*Sal*I-digested pRS413
pRS413-Ykt6-101	Fragment 1	M13 Rv	oTAKA365	pRS416-Ykt6-13	*Bam*HI/*Sal*I-digested pRS413
	Fragment 2	oTAKA364	M13 Fw	pRS416-Ykt6-13	
pRS413-Ykt6-102	Fragment 1	yp1	oTAKA377	genomic DNA	*Bam*HI/*Sal*I-digested pRS413
	Fragment 2	oTAKA376	yp4	pRS416-Ykt6-13	
pRS413-Ykt6-103	Fragment 1	yp1	oTAKA379	pRS416-Ykt6-13	*Bam*HI/*Sal*I-digested pRS413
	Fragment 2	oTAKA378	yp4	genomic DNA	
pRS413-Ykt6-104	Fragment 1	yp1	oTAKA377	genomic DNA	*Bam*HI/*Sal*I-digested pRS413
	Fragment 2	oTAKA376	yp4	pRS416-Ykt6-13	
pRS413-Ykt6-105	Fragment 1	yp1	oTAKA379	pRS413-Ykt6-101	*Bam*HI/*Sal*I-digested pRS413
	Fragment 2	oTAKA378	yp4	genomic DNA	
pRS413-Ykt6-106	Fragment 1	yp1	oTAKA377	pRS416-Ykt6-13	*Bam*HI/*Sal*I-digested pRS413
	Fragment 2	oTAKA376	yp4	genomic DNA	
pRS413-Ykt6-R165Q	Fragment 1	M13 Fw	oSU43	pRS413-Ykt6	*Bam*HI/*Eco*RI-digested pRS413
	Fragment 2	oSU44	M13 Rv	pRS413-Ykt6	
pRS416-3FLAG-Ykt6	Fragment 1	yp1	oTAKA388	genomic DNA	*Sac*I/*Kpn*I-digested pRS416
	Fragment 2	oTAKA387	yp4	genomic DNA	
pRS415-Vam7	Fragment 1	oSU52	oSU53	genomic DNA	*Bam*HI-digested pRS415

Table S5. Continued.

Plasmid name	PCR product used for *in vivo* assembly				Vector DNA used for *in vivo* assembly
		Forward primer	Reverse primer	Template DNA	
pRS415-Vam7-Q284R	Fragment 1	M13 Fw	oSU41	pRS415-Vam7	*Bam*HI/*Hind*III-digested pRS415
	Fragment 2	oSU42	M13 Rv	pRS415-Vam7	
pRS413-Nyv1-R192Q	Fragment 1	M13 Fw	oSU38	pRS413-Nyv1	*Bam*HI/*Eco*RI-digested pRS413
	Fragment 2	oSU37	M13 Rv	pRS413-Nyv1	
pRS416-Sec22	Fragment 1	AK31	AK34	genomic DNA	*Bam*HI/*Hind*III-digested pRS416
pRS416-Sec22-R157Q	Fragment 1	AK31	AK32	genomic DNA	*Bam*HI/*Hind*III-digested pRS416
	Fragment 2	AK33	AK34	genomic DNA	
pRS413-Snc1-R53Q	Fragment 1	M13 Fw	oHW22	pRS413-Snc1	*Sac*I/*Sal*I-digested pRS413
	Fragment 2	oHW21	M13 Rv	pRS413-Snc1	
pCu416-GFP-Vam7	Fragment 1	PNK034	PNK035	pRS413-Vam7	*Eco*RI/*Sal*I-digested pCu416
	Fragment 2	oTAKA252	oTAKA254	pYM44	
pCu416-GFP-Vam7-Q284R	Fragment 1	PNK034	PNK035	pRS413-Vam7-Q284R	*Eco*RI/*Sal*I-digested pCu416
	Fragment 2	oTAKA252	oTAKA254	pYM44	
pCu416-GFP-Sso1	Fragment 1	MY170	MY171	genomic DNA	*Eco*RI/*Kpn*I-digested pCu416
pCu416-GFP-Sso1-Q224R	Fragment 1	M13 Fw	oHW140	pCu416-GFP-Sso1	*Eco*RI/*Bam*HI-digested pRS416
	Fragment 2	oHW141	M13 Rv	pCu416-GFP-Sso1	
pRS423-3FLAG-Ykt6	Fragment 1	M13 Fw	M13 Rv	pRS413-3FLAG-Ykt6	*Xho*I/*Sac*I-digested pRS423
pRS423-3FLAG-Ykt6-R165Q	Fragment 1	M13 Fw	M13 Rv	pRS413-3FLAG-Ykt6-R165Q	*Xho*I/*Sac*I-digested pRS423

Table S6. Plasmid construction by seamless cloning.

Plasmid name	PCR product used for seamless cloning				Vector DNA used for *in vivo* assembly
		Forward primer	Reverse primer	Template DNA	
pRS413-3FLAG-Ykt6-R165Q	Fragment 1	M13 Fw	oSU43	pRS303-3FLAG-Ykt6	*Sac*I/*Xho*I-digested pRS413
	Fragment 2	oSU44	M13 Rv		
pRS413-3FLAG-Snc2	Fragment 1	M13 Fw	oHW99	pRS413-Snc2	*Sac*I/*Xho*I-digested pRS413
	Fragment 2	oHW98	M13 Rv		
pRS413-3FLAG-Snc2-R52Q	Fragment 1	M13 Fw	oHW99	pRS413-Snc2-R52Q	*Sac*I/*Xho*I-digested pRS413
	Fragment 2	oHW98	M13 Rv		
pRS413-3FLAG-Nyv1	Fragment 1	M13 Fw	oHW101	pRS413-Nyv1	*Sac*I/*Xho*I-digested pRS413
	Fragment 2	oHW100	M13 Rv		
pRS413-3FLAG-Nyv1-R192Q	Fragment 1	M13 Fw	oSU38	pRS413-3FLAG-Nyv1	*Sac*I/*Xho*I-digested pRS413
	Fragment 2	oSU37	M13 Rv		

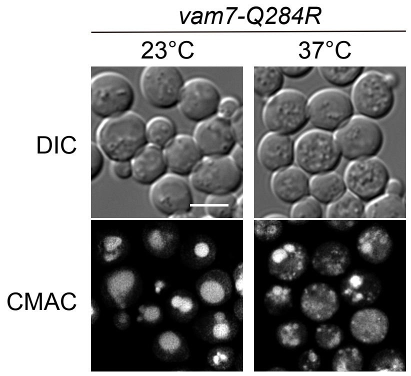

Figure S1. The *vam7-Q284R* strain shows a temperature-sensitive phenotype in terms of vacuole morphology. The *vam7*Δ cells harboring pRS415-Vam7-Q284R and pRS413 were grown in SC−His/Leu medium to the early log phase at 23 °C or 37 °C. The cells were stained with CMAC at the indicated temperatures and observed under a microscope. Scale bar: 5 μm. CMAC, 7-amino-4-chloromethylcoumarin; SC, synthetic complete.

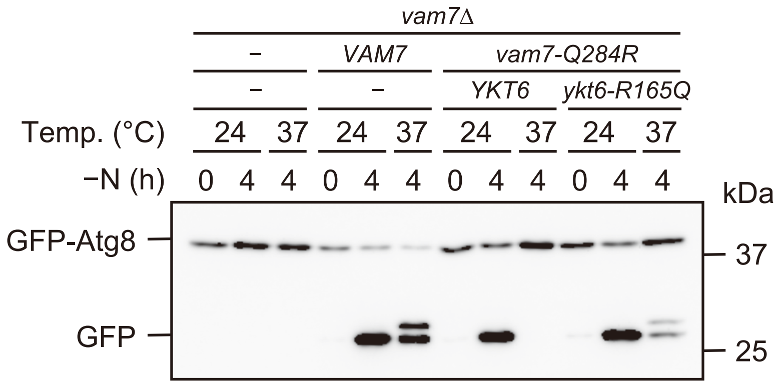

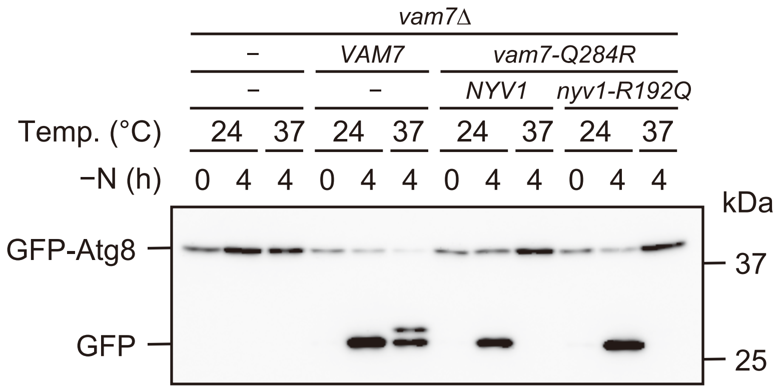

Figure S2. Autophagy of the *vam7-Q284R* mutant strain expressing R-SNARE with a complementary 0-layer mutation. *vam7*Δ (YTS820) cells expressing Vam7-Q284R and either Ykt6, Ykt6-R165Q, Nyv1, or Nyv1-R192Q were grown in SC−His/Leu medium to the early log phase at 24 ℃ and resuspended in SD(−N) medium for further incubation at 24 °C and 37 °C for 4 h. The total cell lysates were subjected to Western blot analysis with anti-GFP antibodies. For the negative and positive control experiments, *vam7*Δ (YTS820) cells transformed with empty vectors (pRS413 and pRS415) and the *VAM7* plasmid (pRS415-Vam7) were used, respectively. SC, synthetic complete; SD, synthetic dextrose.

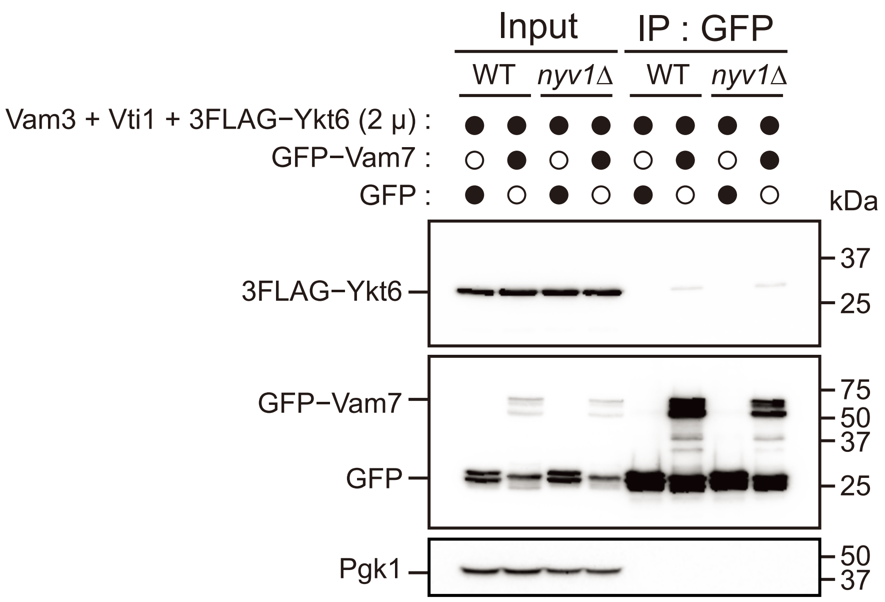

Figure S3. Analysis of physical interactions of Vam7 with Ykt6, by co-immunoprecipitation experiments. Total lysate from the WT (BY4742) and *nyv1*Δ strains overexpressing *VAM3*, *VTI1*, and *3FLAG−YKT6* from a single multicopy plasmid (2 µm plasmid) and either expressing GFP or GFP−Vam7 were used for immunoprecipitation with anti-GFP antibody, and the immunoprecipitates were analyzed by Western blot with anti-FLAG, anti-GFP, and anti-Pgk1 antibodies. Closed circles indicate plasmids retained in the cells used for analyses.

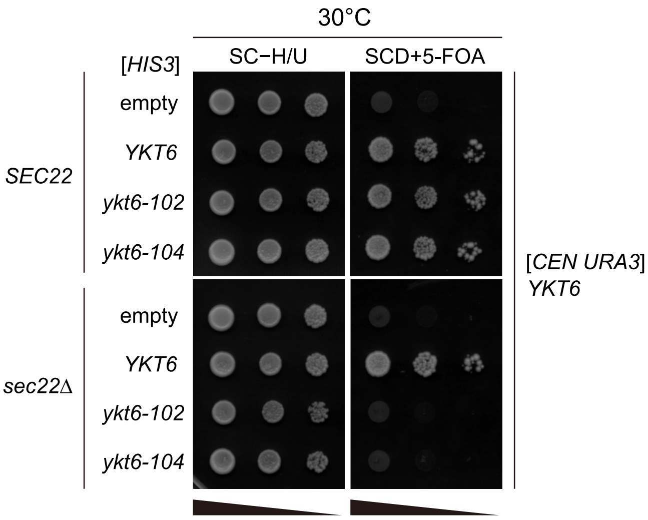

Figure S4. Double mutations (*ykt6* mutation and *sec22* disruption) are lethal in yeast cells.
The *ykt6*Δ (YSU1) and *ykt6*Δ*sec22*Δ (YTS930) strains harboring pAR5 plasmids (*CEN6/ARS URA3 YKT6*) were transformed with plasmids (*CEN6/ARS HIS3*) containing *YKT6*, *ykt6-102*, or *ykt6-104* alleles and grown in SC−His/Ura medium to the early log phase at 30 °C. Cells equivalent to 10^{-2} to 10^{-4} A_{600} units were spotted onto SCD medium containing 5-fluoroorotic acid (5-FOA) and incubated for 3 days at 30 °C to counter-select the cells lacking pAR5 as well as on SC−His/Ura medium as a control experiment. SC, synthetic complete, SCD, synthetic casamino acid dextrose.

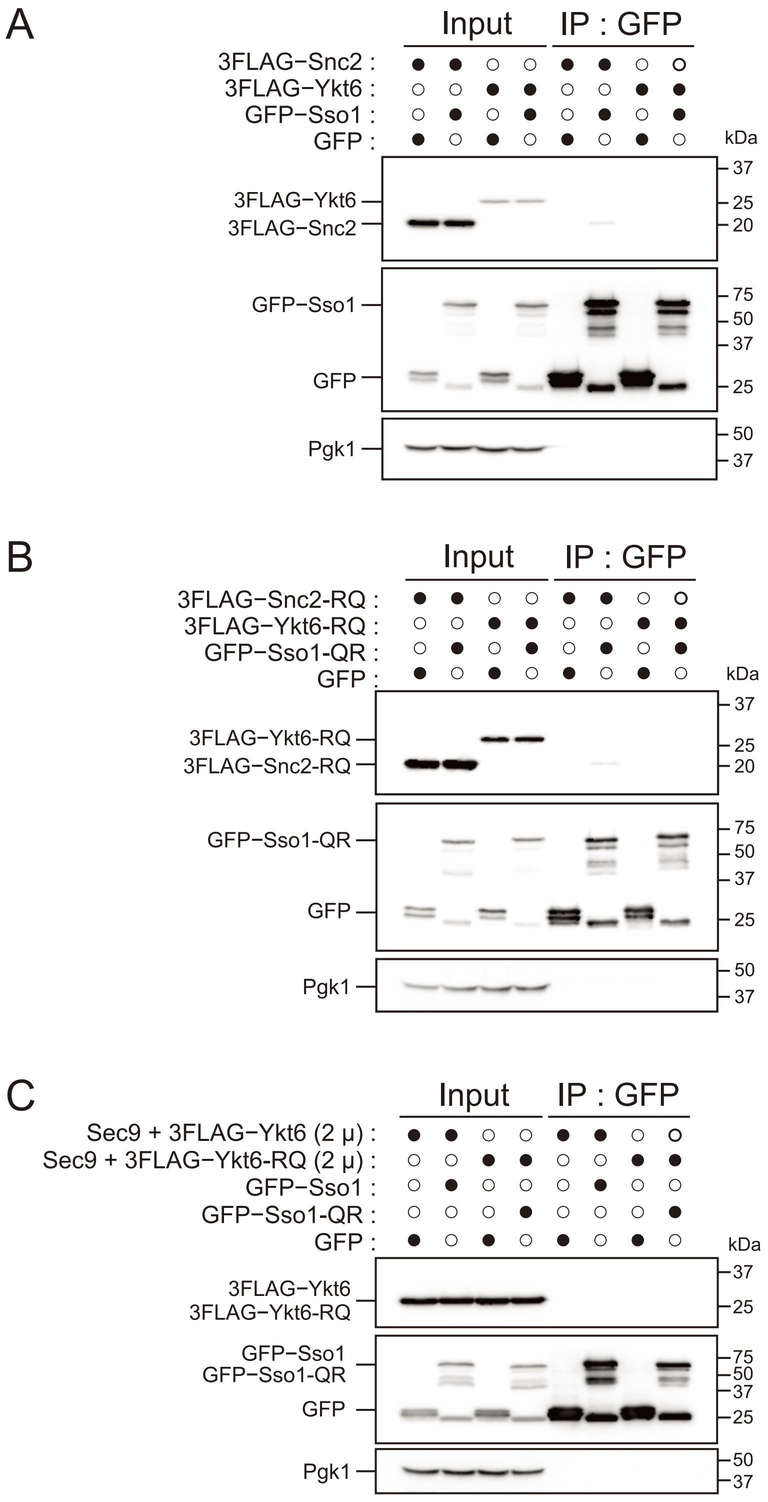

Figure S5. Analysis of physical interactions of Sso1 with Snc2 and Ykt6, by co-immunoprecipitation experiments. *A*, Total lysate from the WT cells (BY4742) expressing either 3FLAG−Snc2 or 3FLAG−Ykt6 from low-copy plasmids and either expressing GFP or GFP−Sso1 were used for immunoprecipitation with anti-GFP antibody, and the immunoprecipitates were analyzed by Western blot with anti-FLAG, anti-GFP, and anti-Pgk1 antibodies. Closed circles indicate plasmids retained in the cells used for analyses. *B*, Total lysate from the WT strains expressing either

3FLAG−Snc2-R52Q or 3FLAG−Ykt6-R165Q from low-copy plasmids and either expressing GFP or GFP−Sso1-Q224R were used for analysis as in *A*. *C*, Co-immunoprecipitation experiments were performed as in *A* and *B* using WT strains overexpressing *SEC9* and *3FLAG−YKT6* or *3FLAG−ykt6-R165Q* from a single multicopy plasmid (2 μm plasmid).

References

1. Liu, A. Y., Koga, H., Goya, C., and Kitabatake, M. (2023) Quick and affordable DNA cloning by reconstitution of Seamless Ligation Cloning Extract using defined factors. *Genes Cells.* **28**, 553-562
2. Mumberg, D., Muller, R., and Funk, M. (1994) Regulatable promoters of *Saccharomyces cerevisiae*: comparison of transcriptional activity and their use for heterologous expression. *Nucleic Acids Res.* **22**, 5767-5768
3. Gnügge, R., Liphardt, T., and Rudolf, F. (2016) A shuttle vector series for precise genetic engineering of *Saccharomyces cerevisiae*. *Yeast.* **33**, 83-98
4. Janke, C., Magiera, M. M., Rathfelder, N., Taxis, C., Reber, S., Maekawa, H. *et al.* (2004) A versatile toolbox for PCR-based tagging of yeast genes: new fluorescent proteins, more markers and promoter substitution cassettes. *Yeast.* **21**, 947-962
5. Shintani, T., Suzuki, K., Kamada, Y., Noda, T., and Ohsumi, Y. (2001) Apg2p functions in autophagosome formation on the perivacuolar structure. *J Biol Chem.* **276**, 30452-30460
6. Kim, J., Huang, W.-P., and Klionsky, D. J. (2001) Membrane recruitment of Aut7p in the autophagy and cytoplasm to vacuole targeting pathways requires Aut1p, Aut2p, and the autophagy conjugation complex. *J Cell Biol.* **152**, 51-64
7. Fujita, S., Sato, D., Kasai, H., Ohashi, M., Tsukue, S., Takekoshi, Y. *et al.* (2018) The C-terminal region of the yeast monocarboxylate transporter Jen1 acts as a glucose signal-responding degron recognized by the alpha-arrestin Rod1. *J Biol Chem.* **293**, 10926-10936
8. Brachmann, C. B., Davies, A., Cost, G. J., Caputo, E., Li, J., Hieter, P. *et al.* (1998) Designer deletion strains derived from *Saccharomyces cerevisiae* S288C: a useful set of strains and plasmids for PCR-mediated gene disruption and other applications. *Yeast.* **14**, 115-132
9. Sikorski, R. S., and Hieter, P. (1989) A system of shuttle vectors and yeast host strains designed for efficient manipulation of DNA in *Saccharomyces cerevisiae*. *Genetics.* **122**, 19-27
10. Kweon, Y., Rothe, A., Conibear, E., and Stevens, T. H. (2003) Ykt6p is a multifunctional yeast R-SNARE that is required for multiple membrane transport pathways to the vacuole. *Mol Biol Cell.* **14**, 1868-1881
11. Katz, L., and Brennwald, P. (2000) Testing the 3Q:1R "rule": mutational analysis of the ionic "zero" layer in the yeast exocytic SNARE complex reveals no requirement for arginine. *Mol Biol Cell.* **11**, 3849-3858
